# Genome-Wide Polygenic Score for Muscle Strength Predicts Risk for Common Diseases and Lifespan: A Prospective Cohort Study

**DOI:** 10.1093/gerona/glae064

**Published:** 2024-03-07

**Authors:** Päivi Herranen, Kaisa Koivunen, Teemu Palviainen, Urho M Kujala, Samuli Ripatti, Jaakko Kaprio, Elina Sillanpää, FinnGen

**Affiliations:** Faculty of Sport and Health Sciences, Gerontology Research Center (GEREC), University of Jyväskylä, Jyväskylä, Finland; Faculty of Sport and Health Sciences, Gerontology Research Center (GEREC), University of Jyväskylä, Jyväskylä, Finland; Institute for Molecular Medicine Finland (FIMM), HiLIFE, University of Helsinki, Helsinki, Finland; Faculty of Sport and Health Sciences, Gerontology Research Center (GEREC), University of Jyväskylä, Jyväskylä, Finland; Institute for Molecular Medicine Finland (FIMM), HiLIFE, University of Helsinki, Helsinki, Finland; Broad Institute of MIT and Harvard, Cambridge, Massachusetts, USA; Institute for Molecular Medicine Finland (FIMM), HiLIFE, University of Helsinki, Helsinki, Finland; Faculty of Sport and Health Sciences, Gerontology Research Center (GEREC), University of Jyväskylä, Jyväskylä, Finland; Wellbeing Services County of Central Finland, Jyväskylä, Finland; Faculty of Sport and Health Sciences, Gerontology Research Center (GEREC), University of Jyväskylä, Jyväskylä, Finland

**Keywords:** FinnGen, Genetics, Hand grip strength, Noncommunicable diseases, Prediction

## Abstract

**Background:**

We used a polygenic score for hand grip strength (PGS HGS) to investigate whether genetic predisposition for higher muscle strength predicts age-related noncommunicable diseases, survival from acute adverse health events, and mortality.

**Methods:**

This study consisted of 342 443 Finnish biobank participants from FinnGen Data Freeze 10 (53% women) aged 40–108 with combined genotype and health registry data. Associations between PGS HGS and a total of 27 clinical endpoints were explored with linear or Cox regression models.

**Results:**

A higher PGS HGS was associated with a reduced risk of selected common noncommunicable diseases and mortality by 2%–10%. The risk for these medical conditions decreased by 5%–23% for participants in the highest PGS HGS quintile compared to those in the lowest PGS HGS quintile. A 1 standard deviation (*SD*) increase in the PGS HGS predicted a lower body mass index (β = −0.112 kg/m^2^, standard error [*SE*] = 0.017, *p* = 1.69E-11) in women but not in men (β = 0.004 kg/m^2^, *p* = .768). PGS HGS was not associated with better survival after acute adverse health events compared to the nondiseased period.

**Conclusions:**

The genotype that supports higher muscle strength appears to protect against future health adversities, albeit with modest effect sizes. Further research is needed to investigate whether or how a favorable lifestyle modifies this intrinsic capacity to resist diseases, and if the impacts of lifestyle behavior on health differs due to genetic predisposition for muscle strength.

Muscle strength may reflect the individual’s intrinsic physiological capacity to resist functional decline into critical disease and disability levels, but also to recover from episodes of poor health over the lifespan (1,2). In particular, low hand grip strength (HGS), measured at any time during adulthood, predicts future adversities, risk of major noncommunicable diseases, and premature mortality (3–9). HGS has also been shown to predict falls (10) and fracture risk (11), and higher HGS assessed before a bone fracture has been observed to be associated with enhanced survival during recovery (12).

Both the HGS and its trajectory over the life course are highly individual and are affected by genes, accumulated lifestyle exposures, the burden of diseases, and progressive physiological aging changes (13,14). Hence, HGS is a multifactorial and polygenic trait. It has a substantial genetic component (heritability estimates of [h^2^] 30%–65%) according to twin studies (15), while genome-wide association studies (GWASs) of HGS have identified a large number of common variants each of a small effect (16,17). No known genetic variants of a large effect on muscle strength have been found (18). Polygenic scores (PGSs) can summarize an individual’s genetic predisposition to a trait into a single value estimate (19). Recently, we constructed a PGS for HGS and showed that it explained 6.1% of the variation in measured HGS and 5.4% of the variation in knee extension strength (20). We also observed that it was associated with better physical functioning, as well as a lower risk of functional limitations among older women. This suggests that the PGS HGS may be used as an estimate of the muscle strength genotype. Individual PGSs can also be used to study genetic pleiotropy, that is, whether the same genetic variation overlaps in 2 or more traits (21). HGS may share a common genetic base with several disease and disability outcomes and subsequent mortality (16,17).

Despite considerable progress in muscle strength research, the genetic aspects of muscle strength are not yet fully understood and might play an important role in healthy aging. We hypothesized that genetically determined muscle strength is an important predictor of future health and lifespan. In this study, we investigated whether PGS HGS predicts common noncommunicable diseases and conditions, and mortality among the Finnish population. Furthermore, the important role of muscle strength in recovering and survival from acute diseases and conditions (2,12) suggests that individuals with a genotype supporting higher muscle strength might have a lower mortality risk after acute adverse health events. To test this hypothesis, we assessed whether the potential association between PGS HGS and mortality risk was pronounced after acute adverse health events compared to the nondiseased period.

## Method

### Study Sample and Endpoints

**Figure 1.**
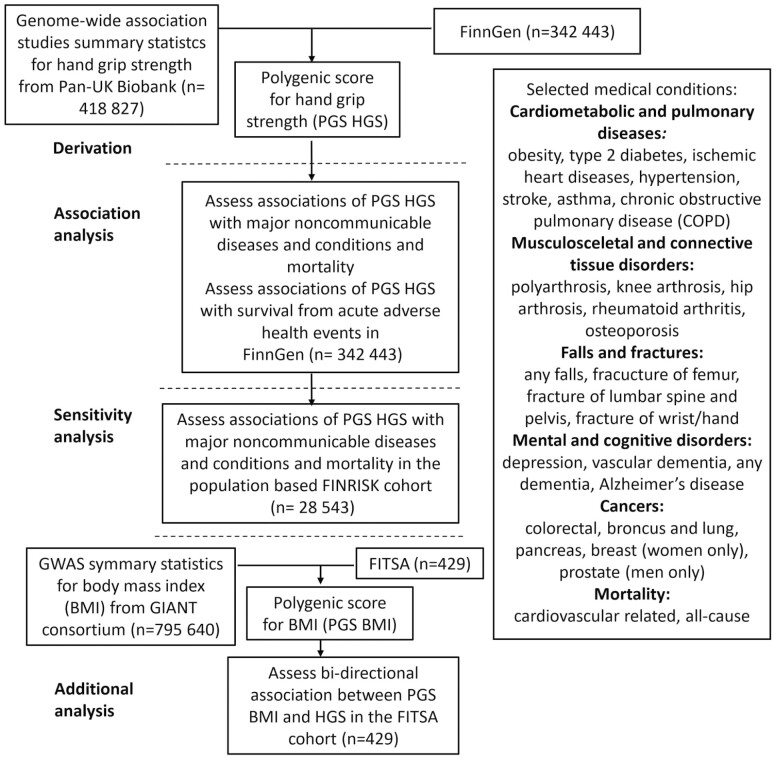
Study design and workflow. The polygenic score for hand grip strength was derived from the Pan-UK Biobank genome-wide association study summary statistics. Association analysis was conducted in the FinnGen cohort of 342 443 Finnish participants and its subcohort FINRISK. Additional analysis was performed in the FITSA cohort. The clinical endpoints used in the analysis were derived from Finnish nationwide digital health registers.

The data comprised 429 200 genotyped Finnish citizens from the latest data freeze 10 of the Finnish FinnGen study (22) (study flow is shown in Figure 1). Genetic principal components (PCs) to correct potential confounding due to population structure (23) were available for 412 181 participants. For this study, we excluded individuals who were under 40 years old at the time of death or at the end of follow-up. The final number of participants included in this analysis was 342 443 individuals. FinnGen includes prospective epidemiological cohorts, disease-based cohorts, and hospital biobank samples (Supplementary Materials; List of FinnGen Data Freeze 10 cohorts). In the FinnGen study, genome information is linked by a unique national personal identification number with national hospital discharge (from 1968), causes of death (from 1969), and cancer (from 1953) registers, and the Social Insurance Institute of Finland (Kela) medication reimbursement (from 1965) and prescribed medicine purchase (from 1995) registers. Endpoint definitions were based on the *International Statistical Classification of Diseases and Related Health Problems* (ICD-8, ICD-9, and ICD-10) codes. In this study, selected endpoints for the analysis were based on the leading causes of death (24) and on the noncommunicable diseases and conditions that are considered major public health issues in Finland (25). The conclusive roster of medical conditions comprised a selection of cardiometabolic and pulmonary diseases, musculoskeletal and connective tissue disorders, falls and fractures, mental and cognitive disorders, cancers, and mortality endpoints (Figure 1).

Endpoint definitions for selected diseases, created by panels of clinical specialists and researchers, are described in the Supplementary Materials; FinnGen endpoint definitions. Detailed descriptions of the ICD codes included in each endpoint can be viewed on the FinnGen website (https://www.finngen.fi/en/researchers/clinical-endpoints, -DF10). In the analysis, adulthood body mass index (BMI) was derived from the health registers, and smoking was categorized into current, former, and never smokers based on self-reports.

The participants provided informed consent for biobank research, based on the Finnish Biobank Act. The ethics approval reference number and other details are given in the Supplementary Materials; Ethical permits of the FinnGen study.

### Genotyping, Quality Control, and Imputation

The FinnGen individuals were genotyped with Illumina and Affymetrix chip arrays (Illumina Inc., San Diego, and Thermo Fisher Scientific, Santa Clara, CA, USA). For detailed information on genotyping, quality control, and imputation, please see Supplementary Materials; Genotyping and quality control of the FinnGen data, and the FinnGen website (https://finngen.gitbook.io/documentation/).

### Polygenic Score for HGS

We adapted a recently developed PGS for maximum HGS (20) to the FinnGen cohort. Briefly, we obtained polygenic scoring by Bayesian methodology (SBayesR) (19) using freely available GWAS summary statistics for maximum HGS from 40- to 69-year-old participants of the Pan-UK Biobank (https://pan.ukbb.broadinstitute.org/). The data were restricted to 418 827 European individuals. The method utilized a sparse linkage disequilibrium (LD) reference panel generated by SBayesR authors. The reference panel was based on a random sample of 50 000 UK Biobank (UKBB) (26) individuals. The original summary statistics included 34 263 104 genetic variants. For computational reasons, the LD reference panel, summary statistics, and FinnGen target study samples were restricted to 1 006 473 HapMap3 (27) variants, which represent the whole genome and are well-imputed for samples of European ancestry. A detailed description of the PGS HGS calculation was presented in our previous study (20).

### Statistical Analyses

#### Association and survival analyses

We analyzed the association between PGS HGS and BMI with linear regression models. We used Cox proportional hazards models to investigate the association between PGS HGS and disease endpoints and mortality. We assessed the proportional hazard assumptions visually by Kaplan–Meier survival curves and by Schoenfeld residuals. We conducted all survival analyses with age as the time scale, and adjusted models for sex, year of DNA sample collection, the first 10 genetic PCs of ancestry, and genotyping batch. Because genetic information and sex remain constant throughout the lifespan of an individual, we set the start of follow-up at birth, and it ended at the first record of the selected endpoint, death, or on December 31, 2021. To avoid possible bias due to left-truncation (28) in survival analysis, we also performed sensitivity analysis by setting the age at the blood sampling for DNA analysis as the start of follow-up, excluding individuals who were affected by the endpoint before DNA sampling. We investigated potential sex differences in the effect of PGS HGS on outcome by fitting the interaction term between PGS HGS and sex into the models. We have presented the results separately for women and men only if we found a significant interaction between the PGS HGS and sex; otherwise, the results are presented adjusted for sex.

#### Time-dependent survival analysis

To analyze mortality risk before and after the onset of an acute adverse health event, we used Cox regression analysis with an extension of the illness–death model (29). We restricted acute adverse health events to ischemic heart diseases, stroke, and femur fracture, as mortality is known to increase during the first year after the event for all these diagnoses (30–32). In the illness–death models, we set the follow-up from 40 years of age because consequences after acute adverse health events are known to be less fatal in the younger population (33). We modeled the disease state as a time-dependent variable in a relative risk model based on a counting process formulation. The possible diseased states of the study participants are shown in Supplementary Figure 1. All participants started in the nondiseased state until an adverse health event occurred, or until death or end of follow-up if they did not have the event of interest. The main effects of PGS HGS indicate mortality risk as PGS changes, and the main effects of diseased states indicate mortality risk compared to nondiseased states. We used interaction terms between PGS HGS and diseased states to investigate whether the association between PGS HGS and mortality risk was different during the first post acute event year or after the first post acute event year compared to the nondiseased state.

#### Sensitivity and additional analysis

It should also be noted that the majority of FinnGen participants have been recruited from hospital biobanks or disease-based cohorts, which may lead to an overestimation of absolute disease risk (34). To check the potential selection bias, we also conducted a sensitivity analysis using a population-based subset of FinnGen, the prospective epidemiological FINRISK study with 28 543 individuals. FINRISK surveys performed in 1992, 1997, 2002, 2007, and 2012 comprised random samples of adults within 5 geographical areas in Finland. Additional details on the study protocol have been described previously (35). In addition, we investigated associations between measured BMI and HGS as well as PGS BMI and HGS in the *Finnish Twin Study on Aging* (36) cohort among 429 Finnish women, aged from 63 to 76 years to be able to observe any bi-directional association (please, see description of the additional analysis in the Supplementary Materials).

In all analyses, we calculated an increase in risk per a 1 standard deviation (*SD*) change in the PGS HGS, and *p*-value < .05 was considered as evidence of an association. To gain further insight into the PGS HGS in the survival analysis, we also divided PGS into 3 groups based on quintiles: low <20%, intermediate 20%–80%, and high >80%. We reported the results as hazard ratios (HRs) together with 95% confidence intervals (CIs) and accounted for multiple testing by controlling the false discovery rate (FDR) at a threshold below 0.05 (37). The FDR correction was calculated by cohorts and separately for each model category, that is, for all basic models, for all interaction models, and separately for men and women according to the results of the Cox regression models. We performed statistical analyses using R 4.2.3 with the R package *survival*, *survminer*, *forestploter*, and *stats*. For the PGS calculation, we used PLINK 2.0 software.

## Results

Table 1 shows the main characteristics of the FinnGen participants. A slight majority (53.2%) were women, and the mean age at the time of death or at the end of follow-up was 66.3 years (range, 40–107.7). Of those with a known smoking status, 47.6% were never smokers.

**Table 1.** Characteristics of Participants in the FinnGen Study

Characteristics	All (*n* = 342 443)	n	Women (*n* = 182 309)	n	Men (*n* = 160 134)	*n*
Mean (*SD*) age (y)	66.29 (12.90)	342 443	64.72 (13.08)	182 309	68.08 (12.47)	160 134
Mean (*SD*) BMI (kg/m^2^)	27.67 (5.30)	247 051	27.73 (5.86)	123 892	27.62 (4.68)	123 159
Mean (*SD*) height (cm)	170.3 (9.14)	248 515	164 (6.31)	124 803	176.7 (6.84)	123 737
Mean (*SD*) weight (kg)	80.49 (17.39)	252 839	74.63 (16.46)	127 189	86.42 (16.27)	125 650
Smoking status *n* (%)		202 771		100 168		102 603
Never	96 553 (47.6)		60 646 (60.5)		35 907 (35.0)	
Former	47 149 (23.3)		20 881 (20.8)		26 268 (25.6)	
Current	59 069 (29.1)		18 641 (18.6)		40 428 (39.4)	

*Notes*: BMI = body mass index; *SD* = standard deviation.

Age at the time of death or at the end of follow-up on December 31, 2021. Phenotype data were obtained from the biobanks (https://www.finngen.fi/en/data_protection/data-protection-statement).

### PGS HGS and Risk for Noncommunicable Diseases and Mortality

**Figure 2.**
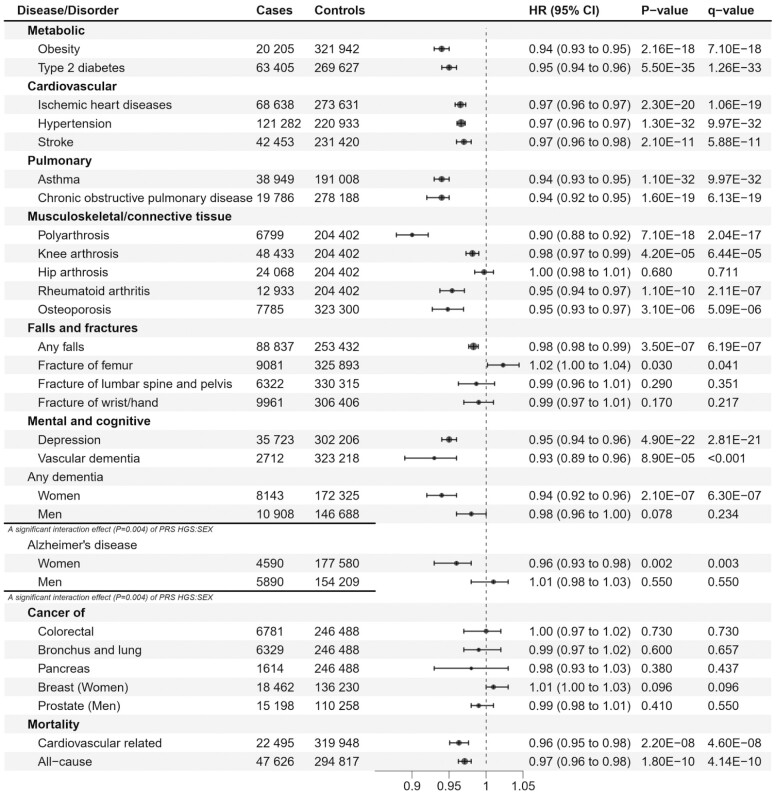
PGS HGS as a predictor of noncommunicable diseases and conditions and mortality in the FinnGen cohort. Multivariable Cox regression analysis. The start of follow-up from birth. Adjusted for sex, year of DNA sample collection, genotyping batch, and 10 genetic principal components of ancestry. CI = confidence Interval, HR = hazard ratio, *q*-value = adjusted *p*-value for the False Discovery Rate (FDR < 0.05).

Participants in the highest PGS HGS quintile demonstrated a noteworthy 5%–23% decreased risk for future health events compared to those in the lowest PGS HGS quintile (risk estimates ranged from 0.95 [0.92–0.97] to 0.77 [0.71–0.83]; Table 2). In general, higher PGS HGS was modestly associated with a cumulative incidence of health adversities (Supplementary Figure 2). As a continuous variable, a 1 *SD* increase in the PGS HGS reduced the risk for polyarthrosis by 10%, for vascular dementia by 7%, obesity diagnosis, asthma, and chronic obstructive pulmonary disease (COPD) by 6%, for type 2 diabetes, rheumatoid arthritis, osteoporosis, and depression by 5%, for death due to cardiovascular causes by 4%, for ischemic heart diseases, hypertension, stroke, and all-cause mortality by 3%, and for knee arthrosis and falls by 2%. HGS was not associated with a risk of hip arthrosis, nor with the risk of fractures or common cancers. HRs with 95% CIs are shown in Figure 2.

**Table 2.** PGS HGS as a Predictor of Common Noncommunicable Diseases and Mortality in the Highest and in the Intermediate Quintiles Compared with the Lowest PGS HGS Quintile

Disease/disorder	An intermediate PGS HGS		A high PGS HGS	
	HR (95 % CI)	*p* Value	HR (95% CI)	*p* Value
Metabolic				
Obesity	0.93 (0.89–0.96)	1.4E-05	0.86 (0.82–0.90)	7.1E-12
Type 2 diabetes	0.94 (0.92–0.96)	1.4E-09	0.87 (0.85–0.89)	1.5E-28
Cardiovascular				
Ischemic heart disease	0.95 (0.93–0.96)	5.9E-09	0.90 (0.88–0.92)	1.3E-18
Hypertension	0.95 (0.94–0.97)	1.7E-10	0.91 (0.89–0.92)	3.6E-27
Stroke	0.95 (0.92–0.97)	1.4E-05	0.91 (0.86–0.94)	2.7E-09
Pulmonary				
Asthma	0.91 (0.89–0.94)	1.2E-12	0.85 (0.83–0.88)	2.4E-23
COPD	0.93 (0.90–0.96)	4.2E-05	0.84 (0.80–0.87)	2.5E-15
Musculoskeletal/connective tissue				
Polyarthrosis	0.87 (0.82–0.92)	4.7E-06	0.77 (0.71–0.83)	5.9E-12
Knee arthrosis	0.98 (0.95–1.00)	0.042	0.95 (0.92–0.97)	1.0E-04
Rheuma	0.94 (0.90–0.98)	6.6E-03	0.88 (0.84–0.93)	9.7E-06
Osteoporosis	0.93 (0.88–0.98)	0.011	0.85 (0.80–0.92)	1.2E-05
Falls	0.98 (0.97–1.00)	0.050	0.94 (0.92–0.96)	7.8E-08
Mental and cognitive				
Depression	0.95 (0.92–0.97)	4.0E-05	0.86 (0.83–0.89)	6.2E-19
Vasculardementia	0.88 (0.80–0.97)	7.7E-03	0.79 (0.70–0.88)	6.7E-05
Any dementia (women)	0.94 (0.89–0.99)	0.029	0.85 (0.80–0.91)	0.71E-06
Alzheimer (women)	0.96 (0.89–1.04)	0.320	0.90 (0.82–0.98)	0.021
Mortality				
Cardiovascular related	0.94 (0.90–0.97)	9.3E-05	0.90 (0.86–0.94)	4.0E-07
All-cause	0.96 (0.94–0.99)	2.4E-03	0.92 (0.89–0.95)	1.2E-08

*Notes*: 95% CI = confidence interval; COPD = Chronic Obstructive Pulmonary Disease; HR = hazard ratio; PGS HGS = polygenic scores for hand grip strength.

Multivariate Cox regression analysis. The start of follow-up from birth. The lowest PGS HGS quintile served as a reference group. Adjusted for sex, genotyping batch, year of DNA sample collection, and 10 genetic principal components of ancestry.

A significant interaction effect between sex and PGS HGS was seen in dementia and Alzheimer’s disease (*P*_interaction_ = .004 for both). Women with a higher PGS HGS had a 6% decreased risk for any dementia and a 4% decreased risk for Alzheimer’s disease, while in men the associations were not statistically significant (Figure 2). In addition, high PGS HGS predicted a lower BMI (β = −0.112 kg·m^−2^, *SE* = 0.017, *p* = 1.69E-11, *n* = 123 878) in women, but not in men (β = 0.004, *SE* = 0.013, *p* = .768, *n* = 123 145, *P*_interaction_ = 2.12E-07 for PGS HGS × SEX).

### Mortality Risk During and After the First Post-Acute Event Year Compared to the Nondiseased Period

The investigated acute adverse health events included ischemic heart disease, stroke, and femur fracture. Supplementary Table 1 shows the characteristics of the participants according to diseased state (nondiseased, survived the first post acute event year, and died during the first post acute event year). The association between PGS HGS and mortality was not pronounced during or after the first post acute year after the acute events compared to the nondiseased period (Supplementary Table 2). The predictive value of PGS HGS on mortality decreased after the first post-stroke year compared to the nondiseased period (Supplementary Tables 1 and 2).

### Sensitivity and Additional Analysis

Supplementary Table 3 shows the characteristics of participants in the FinnGen study when the start of the follow-up was set to the age at the blood sampling for DNA analysis, and characteristics for the FINRISK participants are shown in Supplementary Table 4. Marked differences in results were not observed between the analysis conducted from birth and from blood sampling age or using a population-based FINRISK cohort. However, wide CIs in sensitivity analysis indicated that prognostic imbalance with a small sample size could be substantial (Supplementary Figures 3–5). To investigate the potential bi-directional causality of BMI and HGS, we conducted additional analysis in a subsample of older Finnish women (Supplementary Table 5). We found that PGS BMI did not predict measured HGS (β = −0.746, *SE* = 3.142, *p* = .812).

## Discussion

We utilized a novel genome-wide polygenic scoring methodology and showed that individuals with a genotype supporting higher muscle strength have a reduced risk of several age-related noncommunicable diseases compared with the participants having a genetic predisposition for low muscle strength in a population sample enriched with health care patients. Furthermore, this genotype was associated with a lower risk of mortality due to cardiovascular causes and a lower risk of all-cause mortality, even though the overall gain remained modest. We also investigated the potential role of the PGS HGS during recovery periods and found that genetic predisposition for higher muscle strength did not predict better survival after acute adverse health events. Our results suggest that genetic predisposition for higher muscle strength may reflect an individual’s intrinsic capacity to resist pathological changes that occur over aging, but might not reflect physical resilience, that is, the ability to recover after severe adversity.

Associations between maximal HGS and the occurrence of several noncommunicable conditions, especially cardiometabolic and pulmonary diseases, are well recognized (6–8,38,39). The mechanisms underlying the associations between cardiorespiratory fitness and maximal muscle strength and metabolic risk factors remain unclear but are suggested to be associated with skeletal muscle metabolism, body fat content, and overall metabolic processes (40). Our results suggest that these associations are partly explained by the genetic inheritance of muscle strength and are consistent with large GWASs which have recently succeeded in indicating a partly shared genetic etiology underlying both HGS and common cardiometabolic conditions (16,17). Along with the liver and kidneys, skeletal muscle has a unique ability to store glucose in the form of glycogen, making it the largest metabolic organ and important in maintaining normal blood glucose levels (2). The essential role of skeletal muscle in regulating metabolic homeostasis and respiratory mechanics may contribute to our findings and why genetic predisposition for higher muscle strength protects against cardiometabolic and pulmonary diseases. Our results also advance understanding regarding the partly shared genetic architecture of muscle strength and common cardiopulmonary diseases and highlight the importance of maintaining adequate muscle strength throughout the lifespan.

We found that a genotype that supports higher muscle strength predicted a lower risk of Alzheimer’s disease and dementia in women. These results expand the findings of the latest studies, which have suggested that HGS is associated with early-stage cognitive dysfunctions and all-cause dementia independent of the most important sociodemographic, health, and behavioral confounders (9,41). Furthermore, Tikkanen et al. (16) showed that the HGS genetic score used in their study was significantly associated with cognitive performance, and we recently reported that PGS HGS predicts cognitive tasks in laboratory settings (20). Neuromuscular function underlies maximal muscle strength. Thus, the connection between muscle strength and cognitive performance and disorders might be explained by neurodegenerative and neurochemical changes that affect both phenotypes (42) and/or by shared genetic variations. In our study, a genetic predisposition for higher muscle strength also predicted a lower risk of depression in both sexes. This result is in line with a large study among UKBB participants, which showed that a higher HGS was associated with a lower incidence of depression (43). GWASs have indicated several loci and genes overlap highly with HGS and neuro-developmental disorders or brain function and enrichment of gene expression of brain-related transcripts (16,17). GWASs have also shown genes and gene pathways associated with synaptic structure and neurotransmission but also significant enrichment in the central nervous system and skeletal muscle tissue for variants contributing to the heritability of depressive disorders (44). Based on our results, muscle strength, cognition functions, and depressive disorders may be partly regulated by the same genetic background.

Women and men differ in disease prevalence, manifestation, progression response to treatment, and mortality. At the genetic level, recent studies have found only minor differences in genetic architecture between the sexes in a large number of human traits and diseases (45,46). The disparities in health phenotypes might also be explained by hormonal factors, and differences in physiological characteristics, as well as gender differences in health behavior and sociocultural constructions during the life course (47). In our current study, we observed sex differences in the predictive ability of PGS HGS specifically related to cognition disorders and BMI. BMI is a measure that combines both fat-mass and fat-free mass, including muscle, and these proportions differ by sex at any given BMI value. Studies on the association between HGS and BMI in both genders and all age groups have yielded controversial findings (48,49). Some studies have suggested a genetic correlation between HGS and several measures of body composition such as BMI, lean body mass, body fat, and waist and hip circumference (16,17) as well as causal links between genetic predisposition for adiposity and HGS (50). In our study, we did not find an association between PGS BMI and HGS.

The PGS HGS used in this study is a reliable variable that represents genetic predisposition to overall muscle strength (20). It was derived from GWASs by Pan-UKBB, which were restricted to European ancestry. The Finnish population is known to be a genetic isolate with recent bottlenecks, and the frequency of less common and rare variants differs from that of other Europeans (51). However, the rates of common variants are highly comparable to those of other European populations. It must be noted that, due to UKBB participants being volunteers, they are healthier and may be stronger compared to the general British population and individuals of the same age (52). This suggests that the reported associations in this study may underestimate the true associations. On the other hand, a healthier base population reduces the likelihood that PGS HGS includes genetic variants that are primary predictors of chronic diseases. In the present study, we used a study sample of over 340 000 Finnish individuals over the age of 40 and validated registry-based health care data (53). Our results are well generalizable to Finns and probably Europeans overall because the sample size covers over 11% of the same age in the Finnish population, and sensitivity analyses with population-based FINRISK study suggest similar associations. Minor limitations are that we used existing FinnGen endpoints and did not exclude, for example, violent and accidental deaths or high-energy fractures from our analysis. Second, the FinnGen data set includes register-based phenotypes but offers limited information regarding participants’ lifestyle factors, such as physical activity or exercise. Consequently, this study could not assess the extent to which lifestyle may mediate or moderate the associations between PGS HGS and adverse health events.

## Conclusion

Our results suggest that PGS HGS is a noteworthy predictor of future health adversities among participants representing extreme ends of PGS HGS distribution. PGS HGS may have potential utility alongside traditional risk evaluation in identifying high-risk individuals for common noncommunicable diseases. Or, conversely, genetic factors supporting higher muscle strength may support old-age health. Both high and low PGS interact with and are influenced by other genetic and lifestyle factors influencing individual health outcomes. Therefore, PGS HGS is unlikely to have sufficient clinical utility when used alone. PGS HGS could be applied in further studies to explore whether the associations between muscle strength and future health adversities are causal or are explained by shared genetic and/or environmental factors. In addition, it could be used to study how lifestyle, such as physical activity, modifies human intrinsic capacity to resist diseases and whether their impact on health differs due to genetic predisposition for muscle strength. Further research is also needed to determine whether an individual’s genetic predisposition for muscle strength affects exercise responses and trainability.

## Supplementary Material

glae064_suppl_Supplementary_Figures_S1-S5_Tables_S1-S5

## Data Availability

Researchers can apply to use the FinnGen resource and access the data used. The Finnish biobank data can be accessed through the Fingenious services (https://site.fingenious.fi/en/) managed by FINBB. Finnish Health register data can be applied from Findata (https://findata.fi/en/data/).

## Notes

(Medical Sciences Section)
