# Supplementary material for: Genome-Wide Polygenic Score for Muscle Strength Predicts Risk for Common Diseases and Lifespan: A Prospective Cohort Study
Source: J Gerontol A Biol Sci Med Sci. 2024 Mar 7;79(4):glae064. doi: 10.1093/gerona/glae064 (PMC10972579; doi:10.1093/gerona/glae064)
Supplement: glae064_suppl_Supplementary_Figures_S1-S5_Tables_S1-S5 [file glae064_suppl_supplementary_figures_s1-s5_tables_s1-s5.pdf]

Supplemental Material:

Genome-Wide Polygenic Score for Muscle Strength Predicts Risk for Common Diseases and Lifespan: A Prospective Cohort Study

1. **List of FinnGen Data Freeze 10 Cohorts** 1
2. **FinnGen Endpoint Definitions** 2
3. **Ethical Permits of the FinnGen Study** 9
4. **Genotyping and Quality Control of the FinnGen Data** 10
5. **Illness-Death Model and Results of the Time-Dependent Survival Analysis** 10
6. **Cumulative Incidence Curves from the Main Analysis and Results of the Sensitivity Analysis in the FinnGen Study** 13
7. **Results of the Sensitivity Analysis in the FINRISK Study** 16
8. **Additional Analysis: Description and Results of the Association Analysis Between PGS BMI and HGS for Bi-Directional Associations** 18
9. **References** 18
10. **FinnGen_Banner_Authors** 19

1. List of FinnGen Data Freeze 10 Cohorts

ARCTIC BIOBANK NFBC1966

ARCTIC BIOBANK NFBC1986

AURIA BIOBANK

BIOBANK OF EASTERN FINLAND

BLOOD SERVICE BIOBANK

BOREALIS BIOBANK

CENTRAL FINLAND BIOBANK

HELSINKI BIOBANK

TAMPERE BIOBANK

TERVEYSTALO BIOBANK

THL BIOBANKS:

ATBC

BOTNIA

COROGENE

FINHEALTH 2017

FINHIT

FinIPF

FINRISK 1992-2012

GENERISK

HEALTH 2000/2011

HHS

KUUSAMO (=FR11)

MIGRAINE

SUPER

T1D

FINNISH TWIN COHORT

2. FinnGen Endpoint Definitions

Below are the FinnGen endpoint definitions for selected metabolic, cardiovascular, pulmonary as well as musculoskeletal and connective tissue diseases and cancers, which were used in our analysis. Endpoints are based on the Hospital Discharge registry, the Cause of Death registry, Cancer registry and the Social Insurance Institution of Finland (KELA) registry for reimbursements of medical expenses. More detailed information about the FinnGen endpoints definitions and codes can be found in the FinnGen webpages https://risteys.finngen.fi/ .

Metabolic diseases

Obesity

- Definition: A disorder involving an excessive amount of body fat
- FinnGen code: E4_Obesity
- Hospital Discharge registry & Cause of Death registry:
    - E66

Type 2 diabetes

- Definition: type II diabetes mellitus: A type of diabetes mellitus that is characterized by insulin resistance or desensitization and increased blood glucose levels. This is a chronic disease that can develop gradually over the life of a patient and can be linked to both environmental factors and heredity.
- FinnGen code: T2D
- Include endpoints: T2D_WIDE, E4_DM2

Cardiovascular diseases

Ischemic heart diseases

- Definition: coronary thrombosis: Coagulation of blood in any of the coronary vessels. The presence of a blood clot (thrombus) often leads to myocardial infarction.
- FinnGen code: I9_ISCHHEART
- Include endpoints: I9_ANGINA, I9_MI, I9_MI_STRICT, I9_MI_COMPLICATIONS, I9_POSTAMI, I9_CORATHER, I9_REVASC
- Hospital Discharge registry & Cause of Death registry:
    - ICD-10: 120-125

Hypertension

- Definition: Persistently high systemic arterial blood pressure. Based on multiple readings (blood pressure determination), hypertension is currently defined as when systolic pressure is consistently greater than 140 mm Hg or when diastolic pressure is consistently 90 mm Hg or more.
- FinnGen code: I9_HYPTENS
- Hospital Discharge registry & Cause of Death registry:
    - ICD-10: I10-I15, I67.4

- ICD-9: 4019X|4029A|4029B|4039A|4040A|4059A|4059B|4372A|4059X
- ICD-8: 400|401|402|403|404

Stroke

- Definition: No definition available
- FinnGen code: C_STROKE
- Include endpoints: I9_SAH, I9_ICH, I9_OTHINTRACRA, I9_STR_EXH, I9_STR_SAH, I9_TIA

Pulmonary diseases

COPD

- Definition: chronic obstructive pulmonary disease: A chronic and progressive lung disorder characterized by the loss of elasticity of the bronchial tree and the air sacs, destruction of the air sacs wall, thickening of the bronchial wall, and mucous accumulation in the bronchial tree. The pathologic changes result in the disruption of the air flow in the bronchial airways. Signs and symptoms include shortness of breath, wheezing, productive cough, and chest tightness. The two main types of chronic obstructive pulmonary disease are chronic obstructive bronchitis and emphysema.
- FinnGen code: J10_COPD
- Include endpoints: J10_EMPHYSEMA, J10_COPDNAS

Asthma

- Definition: A bronchial disease that is characterized by chronic inflammation and narrowing of the airways, which is caused by a combination of environmental and genetic factors resulting in recurring periods of wheezing (a whistling sound while breathing), chest tightness, shortness of breath, mucus production and coughing. The symptoms appear due to a variety of triggers such as allergens, irritants, respiratory infections, weather changes, exercise, stress, reflux disease, medications, foods and emotional anxiety.
- FinnGen code: J10_ASTHMA
- Hospital Discharge registry & Cause of Death registry:
    - ICD-10: J45-J46
    - ICD-9: 493
    - ICD-8:493

Musculoskeletal and connective tissue diseases

Arthrosis

- Definition: Hemarthrosis: Bleeding into the joints. It may arise from trauma or spontaneously in patients with hemophilia.
- FinnGen code: M13_ARTHROSIS
- Include endpoints: M13_ARTHROSIS_POLY, M13_ARTHRTOSIS_COX, M13_ARTHROSIS_KNEE, M13_ARTHROSIS_OTH

Polyarthrosis

- Definition: No definition available
- FinnGen code: M13_ARTHROSIS_POLY
- Hospital Discharge registry & Cause of Death registry:
    - ICD-10: M15
    - ICD-9: 7151L|7152L
    - ICD-8: 71303

Knee arthrosis

- Definition: spondyloarthropathy: A group of inflammatory rheumatic diseases associated with arthritis and enthesitis, and often involving the axial skeleton. The most common form of spondyloarthritis is ankylosing spondylitis. Other forms include axial spondyloarthritis, peripheral spondyloarthritis, reactive arthritis, psoriatic arthritis/spondylitis and enteropathic arthritis/spondylitis.
- FinnGen code: M13_ARTHROSIS_KNEE
- Hospital Discharge registry & Cause of Death registry:
    - ICD-10: M17
    - ICD-9: 7151F|7152F
    - ICD-8: 71301

Hip arthrosis

- Definition: osteoarthritis: A noninflammatory degenerative joint disease occurring chiefly in older persons, characterised by degeneration of the articular cartilage, hypertrophy of bone at the margins and changes in the synovial membrane. It is accompanied by pain and stiffness, particularly after prolonged activity.
- FinnGen code: M13_ARTHTROSIS_COX
- Hospital Discharge registry & Cause of Death registry:
    - ICD-10: M16
    - ICD-9: 7151E|7152E
    - ICD-8: 71300

Rheumatoid arthritis

- Definition: rheumatoid arthritis: A chronic, systemic autoimmune disorder characterized by inflammation in the synovial membranes and articular surfaces. It manifests primarily as a symmetric, erosive polyarthritis that spares the axial skeleton and is typically associated with the presence in the serum of rheumatoid factor.
- FinnGen code: M13_RHEUMA
- Hospital Discharge registry & Cause of Death registry:
    - ICD-10: M05, M06
    - ICD-9: 7140A|7140B|7241|7142
    - ICD-8: 712[1-3]

Osteoporosis

- Definition: A condition of reduced bone mass, with decreased cortical thickness and a decrease in the number and size of the trabeculae of cancellous bone (but normal chemical composition), resulting in increased fracture incidence. Osteoporosis is classified as primary (Type 1, postmenopausal osteoporosis; Type 2, age-associated osteoporosis; and idiopathic, which can affect juveniles, premenopausal women, and middle-aged men) and secondary osteoporosis (which results from an identifiable cause of bone mass loss).
- FinnGen code: M13_OSTEOPOROSIS
- Hospital Discharge registry & Cause of Death registry:
  - ICD-10: M80, M81, M82
  - ICD-9: 733[0-1]
  - ICD-8: 7230|72391

Falls and fractures

Falls

- Definition: Falls/tendency to fall
- FinnGen code: FALLS
- Hospital Discharge registry & Cause of Death registry:
  - ICD-10: R29, W00-W19

Femur fracture

- Definition: No definition available.
- FinnGen code: ST19_FRACT_FEMUR
- Hospital Discharge registry & Cause of Death registry:
  - ICD-10: S72
  - ICD-9: 820
  - ICD-8: 820

Fracture of lumbar sacral and pelvis

- Definition: No definition available.
- FinnGen code: ST19_FRACT_LUMBAR_SPINE_PELVIS
- Hospital Discharge registry & Cause of Death registry:
  - ICD-10: S32

Fracture at wrist and hand level

- Definition: No definition available.
- FinnGen code: ST19_FRACT_WRIST_HAND_LEVEL
- Hospital Discharge registry & Cause of Death registry:
  - ICD-10: S62

Mental and cognitive disorders

Depression

- Definition: unipolar depression: A mood disorder having a clinical course involving one or more episodes of serious psychological depression that last two or more weeks each, do not have intervening episodes of mania or hypomania, and are characterized by a loss of interest or pleasure in almost all activities and by some or all of disturbances of appetite, sleep, or psychomotor functioning, a decrease in energy, difficulties in thinking or making decisions, loss of self-esteem or feelings of guilt, and suicidal thoughts or attempts.
- FinnGen code: F5_DEPRESSIO
- Include endpoint: F5_DEPRESSION_PSYCHOTIC
- Hospital Discharge registry & Cause of Death registry:
    - ICD-10: F32, F33
    - ICD-9: 2961|2968|3004

Alzheimer's disease

- Definition: A progressive, neurodegenerative disease characterized by loss of function and death of nerve cells in several areas of the brain leading to loss of cognitive function such as memory and language.
- FinnGen code: G6_ALZHEIMER
- Hospital Discharge registry & Cause of Death registry:
    - ICD-10: G30
    - ICD-9: 3310

Dementia

- Definition: obsolete_dementia: ['An acquired organic mental disorder with loss of intellectual abilities of sufficient severity to interfere with social or occupational functioning. The dysfunction is multifaceted and involves memory, behavior, personality, judgment, attention, spatial relations, language, abstract thought, and other executive functions. The intellectual decline is usually progressive, and initially spares the level of consciousness.']
- FinnGen code: F5_DEMENTIA
- Hospital Discharge registry & Cause of Death registry:
    - ICD-10: F00-F09
    - ICD-9: 290|3310|4378A
    - ICD-8: 290
- KELA reimbursements: Kela codes
    - 307
- Medicine purchases : ATC
    - N06D

Vascular dementia

- Definition: A degenerative vascular disorder affecting the brain. It is caused by the blockage of the blood supply to the brain. It is manifested with decline of memory and cognitive functions.
- FinnGen code: I9_VASCDEM
- Hospital Discharge registry & Cause of Death registry:
  - ICD-10: F01

Cancers

Colorectal cancer (controls excluding all cancers)

- Definition: No definition available.
- FinnGen code: C3_COLORECTAL_EXALLC
- Include endpoints: C3_COLON, C3_RECTOSIGMOID_JUNCTION, C3_RECTUM

Malignant neoplasm of bronchus and lung (controls excluding all cancers)

- Definition: No definition available.
- FinnGen code: C3_BRONCHUS_LUNG_EXALLC
- Cause of Death registry:
  - ICD-10: C34
  - ICD-9: 162
  - ICD-8: 152
  - excluded ICD-9: 1620
  - excluded ICD-8: 1620
- Cancer registry:
  - Topography ICD-O-3 :C34
  - Morphology ICD-O-3: ANY
  - Behaviour codes: 3 (Levels: 0 = "Benign"; 1 = "Semimalignant"; 2 = "Carcinoma in situ"; 3 = "Malignant")

Malignant neoplasm of pancreas (controls excluding all cancers)

- Definition: No definition available.
- FinnGen code: C3_PANCREAS_EXALLC
- Cause of Death registry:
  - ICD-10: C25
  - ICD-9: 157
  - ICD-8: 157
- Cancer registry:

    - Topography ICD-O-3 :C25
    - Morphology ICD-O-3: ANY
    - Behaviour codes: 3

Malignant neoplasm of breast (controls excluding all cancers)

- Definition: No definition available.
- FinnGen code: C3_BREAST_EXALLC
- Cause of Death registry:
    - ICD-10: C50
    - ICD-9: 174
    - ICD-8: 174
- KELA reimbursements: KELA codes:
    - 115
- Cancer registry:
    - Topography ICD-O-3 :C50
    - Morphology ICD-O-3: ANY
    - Behaviour codes: [23]

Malignant neoplasm of prostate (controls excluding all cancers)

- Definition: No definition available.
- FinnGen code: C3_PROSTATE_EXALLC
- Cause of Death registry:
    - ICD-10: C61
    - ICD-9: 185
    - ICD-8: 185
- KELA reimbursements: KELA codes:
    - 116
- Cancer registry:
    - Topography ICD-O-3 :C61
    - Morphology ICD-O-3: ANY
    - Behaviour codes: 3

Mortality

Death due to cardiac causes

- Definition: No definition available.
- FinnGen code: I9_K_CARDIAC

- Cause of Death registry:
    - ICD-10: I00-I02, I05-I09, I10-I15, I20-I25, I26-I28, I30-I52, R96, R98, R99
    - ICD-9: 39|4[0-2]|79[8-9]
    - ICD-8: 39|4[0-2]|79[8-9]

All-cause mortality

- Definition: Any death
- FinnGen code: DEATH
- Cause of Death registry:
    - ICD-10: ANY
    - ICD-9: ANY
    - ICD-8: ANY

3. Ethical Permits of the FinnGen Study

Patients and control subjects in FinnGen and FINRISK provided informed consent for biobank research, based on the Finnish Biobank Act. Alternatively, separate research cohorts, collected prior the Finnish Biobank Act came into effect (in September 2013) and start of FinnGen (August 2017), were collected based on study-specific consents and later transferred to the Finnish biobanks after approval by Fimea (Finnish Medicines Agency), the National Supervisory Authority for Welfare and Health. Recruitment protocols followed the biobank protocols approved by Fimea. The Coordinating Ethics Committee of the Hospital District of Helsinki and Uusimaa (HUS) statement number for the FinnGen study is Nr HUS/990/2017. The FinnGen study is approved by Finnish Institute for Health and Welfare (permit numbers: THL/2031/6.02.00/2017, THL/1101/5.05.00/2017, THL/341/6.02.00/2018, THL/2222/6.02.00/2018, THL/283/6.02.00/2019, THL/1721/5.05.00/2019 and THL/1524/5.05.00/2020), Digital and population data service agency (permit numbers: VRK43431/2017-3, VRK/6909/2018-3, VRK/4415/2019-3), the Social Insurance Institution (permit numbers: KELA 58/522/2017, KELA 131/522/2018, KELA 70/522/2019, KELA 98/522/2019, KELA 134/522/2019, KELA 138/522/2019, KELA 2/522/2020, KELA 16/522/2020), Findata permit numbers THL/2364/14.02/2020, THL/4055/14.06.00/2020, THL/3433/14.06.00/2020, THL/4432/14.06/2020, THL/5189/14.06/2020, THL/5894/14.06.00/2020, THL/6619/14.06.00/2020, THL/209/14.06.00/2021, THL/688/14.06.00/2021, THL/1284/14.06.00/2021, THL/1965/14.06.00/2021, THL/5546/14.02.00/2020, THL/2658/14.06.00/2021, THL/4235/14.06.00/2021, Statistics Finland (permit numbers: TK-53-1041-17 and TK/143/07.03.00/2020 (earlier TK-53-90-20) TK/1735/07.03.00/2021, TK/3112/07.03.00/2021) and Finnish Registry for Kidney Diseases permission/extract from the meeting minutes on 4th July 2019. The Biobank Access Decisions for FinnGen samples and data utilized in FinnGen Data Freeze 10 include: THL Biobank BB2017_55, BB2017_111, BB2018_19, BB_2018_34, BB_2018_67, BB2018_71, BB2019_7, BB2019_8, BB2019_26, BB2020_1, BB2021_65, Finnish Red Cross Blood Service Biobank 7.12.2017, Helsinki Biobank HUS/359/2017, HUS/248/2020, HUS/150/2022 § 12, §13, §14, §15, §16, §17, §18, and §23, Auria Biobank AB17-5154 and amendment #1 (August 17 2020) and amendments BB_2021-0140, BB_2021-0156 (August 26 2021, Feb 2 2022), BB_2021-0169, BB_2021-0179, BB_2021-0161, AB20-5926 and amendment #1 (April 23 2020)and it´s modification (Sep 22 2021), Biobank Borealis of Northern Finland_2017_1013, 2021_5010, 2021_5018, 2021_5015, 2021_5023, 2021_5017, 2022_6001, Biobank of Eastern Finland 1186/2018 and amendment 22 § /2020, 53§/2021, 13§/2022, 14§/2022, 15§/2022, Finnish Clinical Biobank Tampere MH0004 and amendments (21.02.2020 & 06.10.2020), §8/2021, §9/2022, §10/2022, §12/2022, §20/2022, §21/2022, §22/2022, §23/2022, Central Finland Biobank 1-2017, and Terveystalo Biobank STB 2018001 and amendment 25th Aug 2020, Finnish Hematological Registry and Clinical Biobank decision 18th June 2021, Arctic biobank P0844: ARC_2021_1001.

4. Genotyping and Quality Control of the FinnGen Data

Chip genotyping was done using several Illumina and Affymetrix FinnGen Axiom arrays. The algorithms for genotype calling were GenCall or GenCall+zCall for Illumina and AxiomGT1 for Affymetrix chip genotypes. The genome build of all genotypes was set to GRCh38/hg38. Quality control exclusions were done sample-wise: samples with a call rate below 95% and heterozygosity test method-of-moments F coefficient estimate value deviated more than ±4SD from the mean were removed along with the samples which failed sex check or were among the multi-dimensional scaling principal component analysis outliers, and in variant-wise: variants with a call rate below 98%, minor allele count below 3 and Hardy-Weinberg Equilibrium p-value lower than 1e-06 were removed.

Pre-phasing was performed using Eagle v2.3.5 and imputation with Beagle v4.1 (protocol described in dx.doi.org/10.17504/protocols.io.xbgfijw) using Sisu v4 as reference panel which consists of 8,554 Finnish whole genome sequences (depth up to 30x). As a post-imputation quality control, variants with imputation quality score below 0.7 were removed.

5. Illness-Death Model and Results of the Time-Dependent Survival Analysis

eFigure 1.

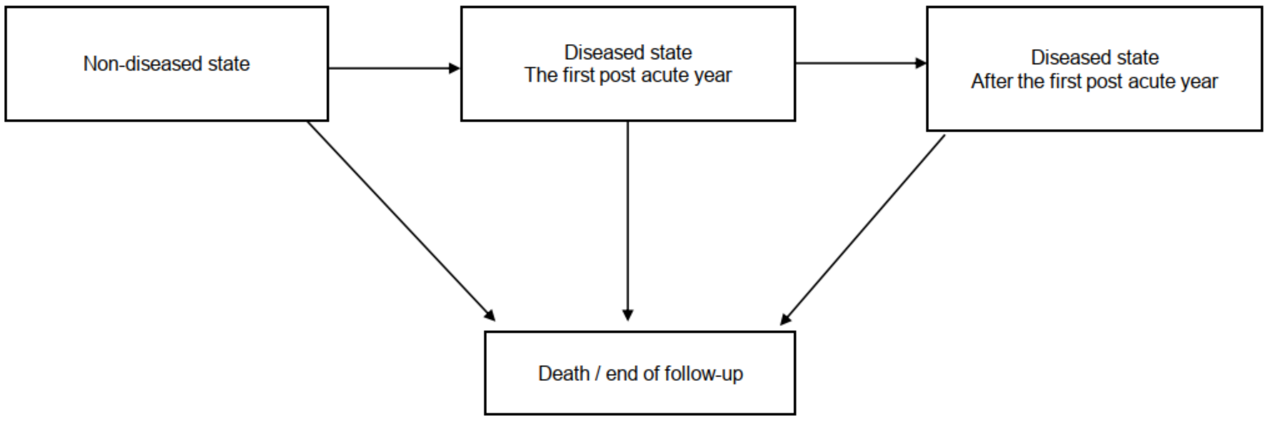

An extension of the illness–death model used in the analysis.

eTable 1 shows the characteristics of the participants according to diseased state (non-diseased, survived the first post acute event year, and died during the first post acute event year). In total, 9 992 (14.8%) participants who faced acute ischemic heart disease and 5 877 (14.4%) participants with stroke died during the first year after the event. Of the participants with femur fracture, 2 007 (30.8%) died during the first year post-fracture. In all participants, those who died during the first year after an acute event were significantly older and were likely to be former or current smokers compared to the first-year post acute event survivors and non-diseased participants.

eTable 1. Characteristics of FinnGen Participants Stratified into Those Who Did Not Sustain an Acute Event (Non-Diseased), Those Who Sustained an Acute Event and Either Survived the first Post Acute Year (Diseased, Survived) or Died During the First Post Acute Event Year (Diseased, Died During the First Year)

	Non-diseased	Diseased, survived the first year	Diseased, died during the first year	P*
Ischemic heart disease n	273 756	57 358	9992	
PGS HGS mean (SD)	0.005 (1.00)	-0.023 (1.00)	-0.006 (1.00)	<0.001
Age at baseline mean (SD)	64.0 (12.5)	64.0 (10.7)	73.5 (10.3)	<0.001§
BMI mean (SD)	27.6 (5.41)	28.0 (4.86)	27.5 (4.84)	<0.001§
Current smokers n (%)	41 168 (25.9)	13 225 (36.7)	4397 (61.4)	<0.001ᐩ
Stroke N	231 511	34 896	5877	
PGS HGS mean (SD)	0.008 (1.00)	-0.016 (1.01)	0.016 (1.00)	<0.001
Age at baseline mean (SD)	62.8 (12.2)	65.7 (11.4)	74.6 (10.6)	<0.001§
BMI mean (SD)	27.5 (5.37)	27.6 (4.84)	27.1 (4.60)	<0.001§
Current smokers n (%)	32 413 (24.1)	7935 (35.8)	1955 (49.2)	<0.001ᐩ
Femur fracture n	326 052	6507	2007	
PGS HGS mean (SD)	-0.001 (1.00)	0.049 (1.01)	0.046 (1.00)	<0.001
Age at baseline mean (SD)	65.9 (12.8)	71.7 (12.4)	80.2 (9.19)	<0.001§
BMI mean (SD)	27.7 (5.31)	26.5 (4.85)	26.6 (4.65)	<0.001§
Current smokers n (%)	55 247 (28.7)	1821 (42.5)	777 (53.7)	<0.001ᐩ

Note: *One-way analysis of variance, §Welch test for variables with unequal variances between groups, ᐩChi square test, PGS HGS = Polygenic Scores for Hand Grip Strength, SD=Standard Deviation, BMI= Body Mass Index.

eTable 2. Main Effects and Interactions of PGS HGS and Diseased State on the Mortality Risk in the FinnGen Cohort

Phenotype	HR (95% CI)	P
Ischemic heart disease (n=341 106)		
PGS HGS	**0.97 (0.96–0.99)**	**2.3 x 10^{-05}**
Diseased state		
Non-diseased state	1.00	
The first post acute year	**4.71 (4.53–4.90)**	**<0.001**
After the first post acute year	**1.70 (1.67–1.74)**	**<0.001**
Sex		
Women	1.00	
Men	**1.82 (1.78–1.86)**	**<0.001**
PGS HGS and diseased state		
PGS HGS*Non-diseased state	1.00	
PGS HGS*The first post acute year	1.00 (0.96–1.04)	0.940
PGS HGS*After the first post acute year	1.00 (0.98–1.02)	0.960
Stroke (n=272 284)		
PGS HGS	**0.98 (0.97–0.99)**	**5.5 x 10^{-05}**
Diseased state		
Non-diseased state	1.00	
The first post acute year	**5.48 (5.27–5.70)**	**<0.001**
After the first post acute year	**1.80 (1.76–1.84)**	**<0.001**
Sex		
Women	1.00	
Men	**1.89 (1.85–1.93)**	**<0.001**
PGS HGS and diseased state		
PGS HGS*Non-diseased state	1.00	
PGS HGS*The first post acute year	1.02 (0.98–1.06)	0.260
PGS HGS*After the first post acute year	**0.97 (0.95–0.99)**	**0.004**
Femur fracture (n=334 566)		
PGS HGS	**0.97 (0.96–0.98)**	**1.8 x 10^{-10}**
Diseased state		
Non-diseased state	1.00	
The first post acute year	**5.54 (5.24–5.87)**	**<0.001**
After the first post acute year	**2.22 (2.13–5.87)**	**<0.001**
Sex		
Women		
Men	**1.98 (1.93–2.02)**	**<0.001**
PGS HGS and diseased state		
PGS HGS*Non-diseased state	1.00	
PGS HGS*The first post acute year	1.00 (0.95–1.06)	0.950
PGS HGS*After the first post acute year	0.99 (0.95–1.03)	0.520

Note: Extended Cox regression analysis. Reference group: sex, women; non-diseased state (no acute adverse health event during the follow-up and time before an acute event occurrence of the participants sustaining an acute event). Adjusted for sex, year of DNA sample collection, genotyping batch, and ten genetic principal components of ancestry. PGS HGS = Polygenic Scores for Hand Grip Strength, HR=Hazard Ratio, CI=Confidence Interval. Statistically significant values are shown in bold.

6. Cumulative Incidence Curves from the Main Analysis and Results of the Sensitivity Analysis in the FinnGen Study

eFigure 2.

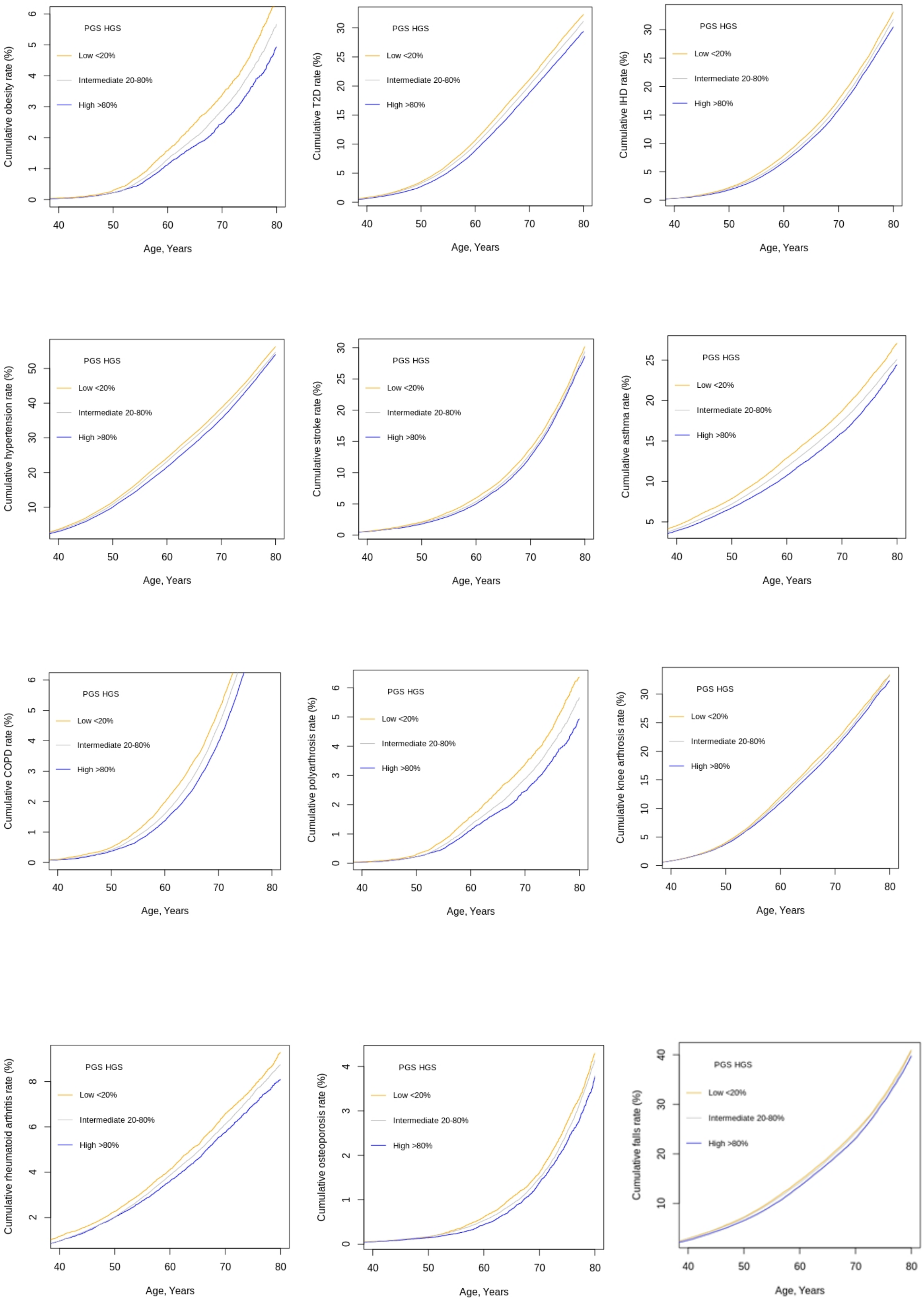

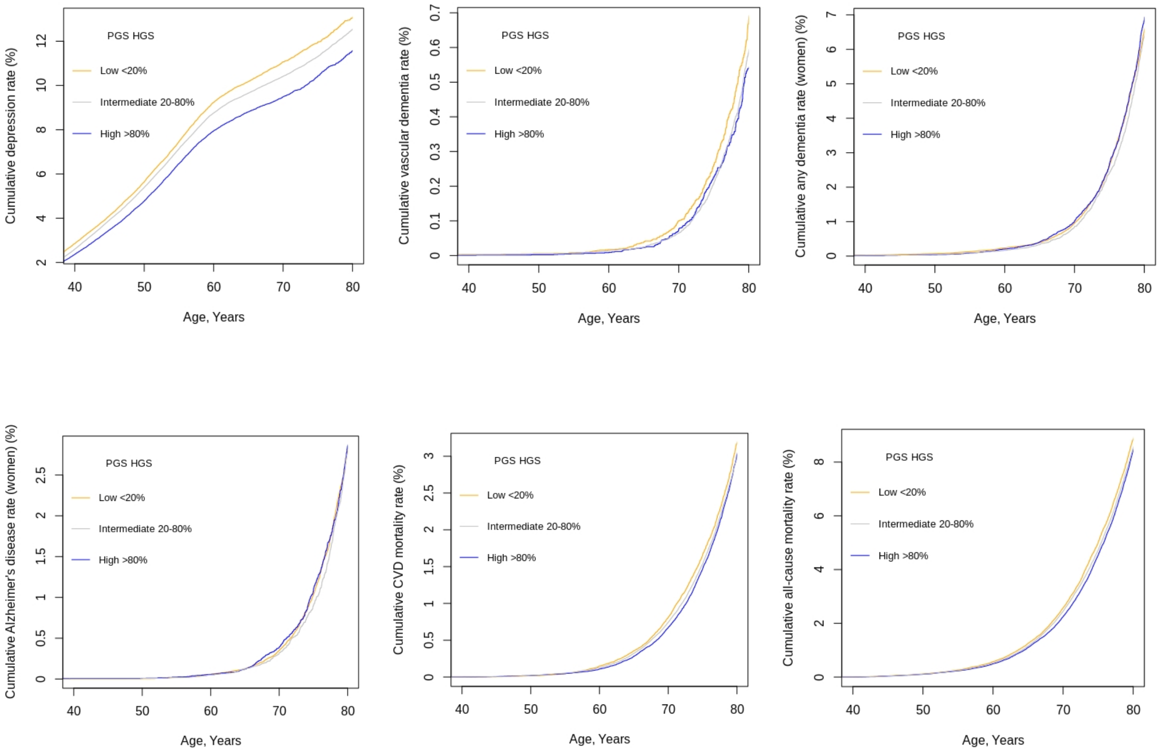

Cumulative incidence of diseases and mortality by PGS HGS categories in FinnGen for the scaled age. Cumulative incidence is presented as a percentage. The survival curves are from the multivariable Cox regression analysis. The start of follow-up from birth**.** Only endpoints, which provided statistical significance are presented. Adjusted for sex, year of DNA sample collection, genotyping batch, and ten genetic principal components of ancestry.

eTable 3. Characteristics of Participants in the FinnGen Study When Start of the Follow-Up Was Set to the Age at the Blood Sampling for DNA Analysis

Characteristics	All (n=339 983)	n	Women (n=180 926)	n	Men (n=159 057)	n
Mean (SD) age (y)	66.30 (12.90)	339 983	64.72 (13.07)	180 926	68.09 (12.47)	159 057
Mean (SD) BMI (kg/m^2)	27.67 (5.30)	245 637	27.72 (5.85)	123 128	27.62 (4.68)	122 499
Mean (SD) height (cm)	170.3 (9.14)	247 095	164.00 (6.31)	124 043	176.70 (6.84)	123 052
Mean (SD) weight (kg)	80.48 (17.38)	251 359	74.62 (16.45)	126 389	86.40 (16.26)	124 970
Smoking status n (%)		201 896		99 701		102 195
Never	96 036 (47.60)		60 327 (60.50)		35 709 (34.90)	
Former	46 979 (23.30)		20 820 (20.88)		26 159 (25.60)	
Current	58 881 (29.20)		18 554 (18.60)		40 327 (39.46)	

Note: Age at the time of death or at the end of follow-up on 31 December 2021. Phenotype data were obtained from the biobanks https://www.finngen.fi/en/data_protection/data-protection-statement), SD=Standard Deviation, BMI=Body Mass Index.

eFigure 3.

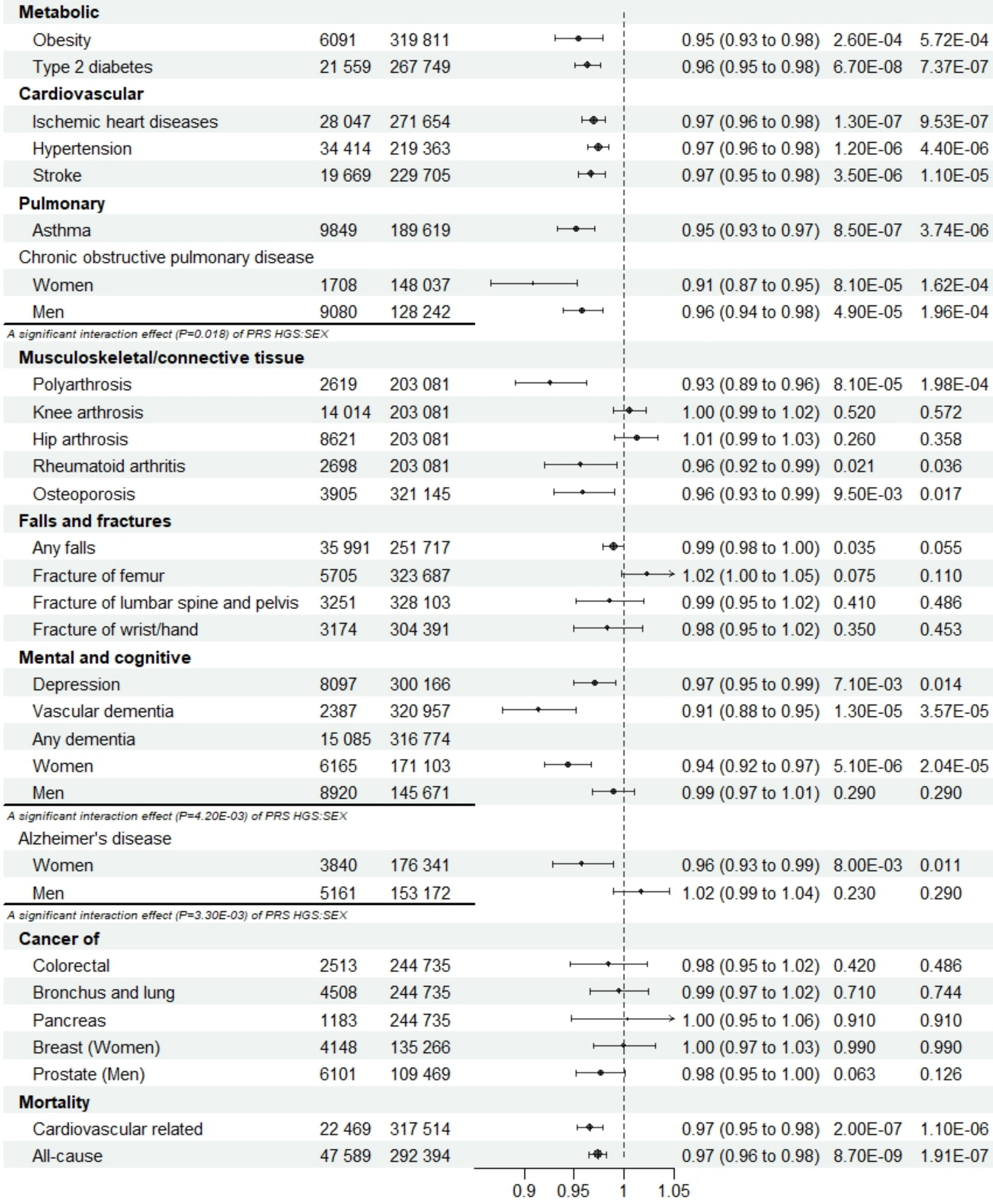

Disease/Disorder	Cases	Controls	HR (95% CI)	P-value	q-value
**Metabolic**					
Obesity	6091	319 811	0.95 (0.93 to 0.98)	2.60E-04	5.72E-04
Type 2 diabetes	21 559	267 749	0.96 (0.95 to 0.98)	6.70E-08	7.37E-07
**Cardiovascular**					
Ischemic heart diseases	28 047	271 654	0.97 (0.96 to 0.98)	1.30E-07	9.53E-07
Hypertension	34 414	219 363	0.97 (0.96 to 0.98)	1.20E-06	4.40E-06
Stroke	19 669	229 705	0.97 (0.95 to 0.98)	3.50E-06	1.10E-05
**Pulmonary**					
Asthma	9849	189 619	0.95 (0.93 to 0.97)	8.50E-07	3.74E-06
Chronic obstructive pulmonary disease					
Women	1708	148 037	0.91 (0.87 to 0.95)	8.10E-05	1.62E-04
Men	9080	128 242	0.96 (0.94 to 0.98)	4.90E-05	1.96E-04
A significant interaction effect (P=0.018) of PRS HGS:SEX					
**Musculoskeletal/connective tissue**					
Polyarthrosis	2619	203 081	0.93 (0.89 to 0.96)	8.10E-05	1.98E-04
Knee arthrosis	14 014	203 081	1.00 (0.99 to 1.02)	0.520	0.572
Hip arthrosis	8621	203 081	1.01 (0.99 to 1.03)	0.260	0.358
Rheumatoid arthritis	2698	203 081	0.96 (0.92 to 0.99)	0.021	0.036
Osteoporosis	3905	321 145	0.96 (0.93 to 0.99)	9.50E-03	0.017
**Falls and fractures**					
Any falls	35 991	251 717	0.99 (0.98 to 1.00)	0.035	0.055
Fracture of femur	5705	323 687	1.02 (1.00 to 1.05)	0.075	0.110
Fracture of lumbar spine and pelvis	3251	328 103	0.99 (0.95 to 1.02)	0.410	0.486
Fracture of wrist/hand	3174	304 391	0.98 (0.95 to 1.02)	0.350	0.453
**Mental and cognitive**					
Depression	8097	300 166	0.97 (0.95 to 0.99)	7.10E-03	0.014
Vascular dementia	2387	320 957	0.91 (0.88 to 0.95)	1.30E-05	3.57E-05
Any dementia	15 085	316 774			
Women	6165	171 103	0.94 (0.92 to 0.97)	5.10E-06	2.04E-05
Men	8920	145 671	0.99 (0.97 to 1.01)	0.290	0.290
A significant interaction effect (P=4.20E-03) of PRS HGS:SEX					
Alzheimer's disease					
Women	3840	176 341	0.96 (0.93 to 0.99)	8.00E-03	0.011
Men	5161	153 172	1.02 (0.99 to 1.04)	0.230	0.290
A significant interaction effect (P=3.30E-03) of PRS HGS:SEX					
**Cancer of**					
Colorectal	2513	244 735	0.98 (0.95 to 1.02)	0.420	0.486
Bronchus and lung	4508	244 735	0.99 (0.97 to 1.02)	0.710	0.744
Pancreas	1183	244 735	1.00 (0.95 to 1.06)	0.910	0.910
Breast (Women)	4148	135 266	1.00 (0.97 to 1.03)	0.990	0.990
Prostate (Men)	6101	109 469	0.98 (0.95 to 1.00)	0.063	0.126
**Mortality**					
Cardiovascular related	22 469	317 514	0.97 (0.95 to 0.98)	2.00E-07	1.10E-06
All-cause	47 589	292 394	0.97 (0.96 to 0.98)	8.70E-09	1.91E-07

PGS HGS as a predictor of noncommunicable diseases and conditions and mortality in the FinnGen cohort. Multivariable Cox regression analysis. The start of follow-up from age at baseline data collection, which is also the blood sampling for DNA analysis. Adjusted for sex, year of DNA sample collection, genotyping batch, and ten genetic principal components of ancestry. HR=Hazard Ratio, CI=Confidence Interval, q-value = adjusted p-value for the False Discovery Rate (FDR <0.05).

7. Results of the Sensitivity Analysis in the FINRISK Study

eTable 4. Characteristics of Participants in the Population-Based FINRISK Study

Characteristics	All (n=28 543)	n	Women n=15 138)	n	Men (n=13 405)	n
Mean (SD) age (y)	67.63 (12.34)	28 543	67.54 (12.57)	15 138	67.73 (12.09)	13 405
Mean (SD) BMI (kg/m^2)	26.83 (4.70)	28 448	26.50 (5.12)	15 118	27.21 (4.14)	13 330
Mean (SD) height (cm)	168.80 (9.32)	28 452	162.60 (6.26)	15 120	175.90 (6.86)	13 332
Mean (SD) weight (kg)	76.65 (15.40)	28 449	69.97 (13.52)	15 119	84.22 (13.81)	13 330
Smoking status n (%)		27 449		14 646		12 803
Never	14 645 (53.35)		9409 (64.24)		5236 (40.90)	
Former	6267 (22.83)		2461 (16.80)		3806 (29.73)	
Current	6537 (23.82)		2776 (18.95)		3761 (29.38)	

Note: Age at the time of death or at the end of follow-up on 31 December 2021. Phenotype data were obtained from the THL Biobank (https://thl.fi/en/web/thlfi-en/research-and-development/research-and-projects/the-national-finrisk-study), SD=Standard Deviation, BMI=Body Mass Index.

eFigure 4.

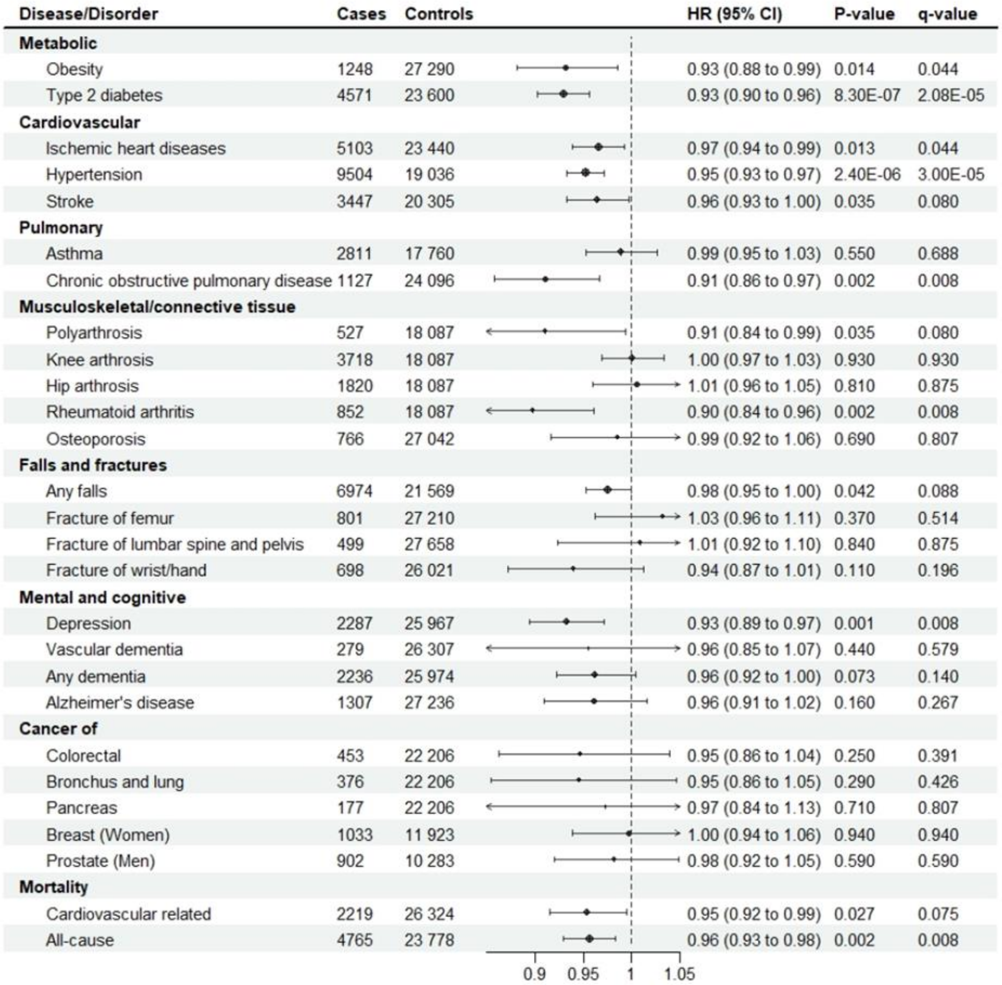

PGS HGS as a predictor of noncommunicable diseases and conditions and mortality in the FINRISK cohort. Multivariable Cox regression analysis. The start of follow-up from birth. Adjusted for sex, year of DNA sample collection, genotyping batch, and ten genetic principal components of ancestry. HR=Hazard Ratio, CI=Confidence Interval, q-value = adjusted p-value for the False Discovery Rate (FDR <0.05).

eFigure 5.

Disease/Disorder	Cases	Controls	HR (95% CI)	P-value	q-value
**Metabolic**					
Obesity	960	27 290	0.95 (0.89 to 1.02)	0.140	0.350
Type 2 diabetes	3685	23 600	0.95 (0.92 to 0.98)	1.40E-03	0.012
**Cardiovascular**					
Ischemic heart diseases	3867	23 440	0.99 (0.96 to 1.02)	0.430	0.566
Hypertension	5879	19 036	0.96 (0.93 to 0.98)	1.00E-03	0.012
Stroke	2825	20 305	0.98 (0.94 to 1.02)	0.280	0.500
**Pulmonary**					
Asthma	1733	17 760	0.98 (0.93 to 1.03)	0.390	0.556
Chronic obstructive pulmonary disease	967	24 096	0.89 (0.84 to 0.95)	5.00E-03	0.012
**Musculoskeletal/connective tissue**					
Polyarthrosis	467	18 087	0.92 (0.84 to 1.01)	0.082	0.256
Knee arthrosis	2774	18 087	1.01 (0.98 to 1.05)	0.490	0.583
Hip arthrosis	1502	18 087	1.01 (0.96 to 1.06)	0.780	0.780
Rheumatoid arthritis	493	18 087	0.94 (0.86 to 1.03)	0.170	0.375
Osteoporosis	710	27 042	0.98 (0.91 to 1.05)	0.570	0.648
**Falls and fractures**					
Any falls	5910	21 569	0.98 (0.95 to 1.00)	0.120	0.333
Fracture of femur	693	27 210	1.02 (0.95 to 1.10)	0.610	0.663
Fracture of lumbar spine and pelvis	448	27 658	1.03 (0.94 to 1.13)	0.490	0.583
Fracture of wrist/hand	546	26 021	0.96 (0.88 to 1.04)	0.330	0.516
**Mental and cognitive**					
Depression	1613	25 967	0.95 (0.91 to 1.00)	0.056	0.233
Vascular dementia	268	26 307	0.94 (0.83 to 1.06)	0.300	0.500
Any dementia	2208	25 974	0.96 (0.92 to 1.00)	0.073	0.256
Alzheimer's disease	1300	27 236	0.96 (0.91 to 1.02)	0.180	0.375
**Cancer of**					
Colorectal	389	22 206	0.95 (0.86 to 1.05)	0.290	0.500
Bronchus and lung	357	22 206	0.96 (0.86 to 1.06)	0.400	0.556
Pancreas	174	22 206	0.97 (0.83 to 1.12)	0.680	0.708
Breast (Women)	777	11 923	0.96 (0.89 to 1.03)	0.230	0.230
Prostate (Men)	792	10 283	0.97 (0.91 to 1.04)	0.440	0.440
**Mortality**					
Cardiovascular related	2219	26 324	0.96 (0.92 to 1.00)	0.032	0.160
All-cause	4765	23 778	0.96 (0.93 to 0.98)	2.60E-03	0.016

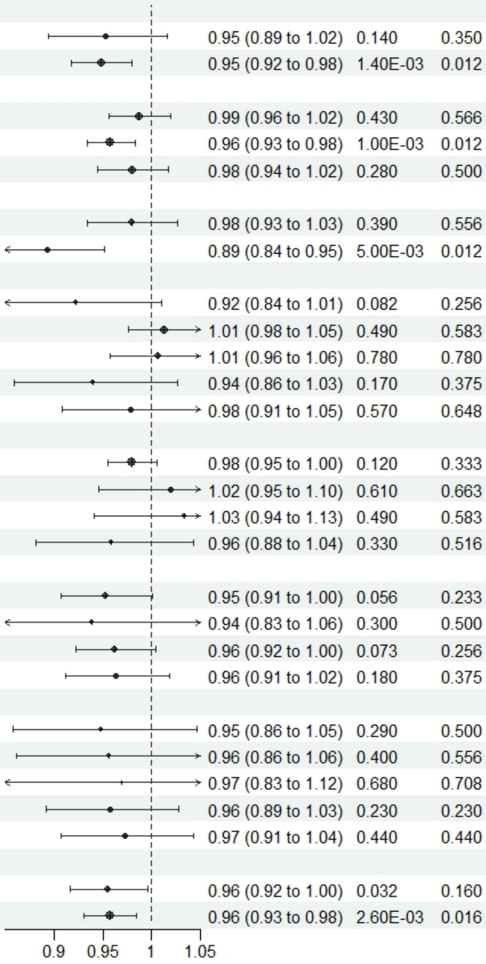

PGS HGS as a predictor of noncommunicable diseases and conditions and mortality in the FINRISK cohort. Multivariable Cox regression analysis. The start of follow-up from age at baseline data collection, which is also the blood sampling for DNA analysis. Adjusted for sex, year of DNA sample collection, genotyping batch, and ten genetic principal components of ancestry. HR=Hazard Ratio, CI=Confidence Interval, q-value = adjusted p-value for the False Discovery Rate (FDR <0.05.

8. Additional Analysis: Description and Results of the Association Analysis Between PGS BMI and HGS for Bi-Directional Associations

We investigated associations between measured BMI and HGS as well as PGS BMI and HGS in the *Finnish Twin Study on Aging* (FITSA) (1) cohort among 429 Finnish women, aged from 63 to 76 years (eTable 5) to be able to observe any bi-directional association. Polygenic scoring was conducted using GWAS meta-analysis summary statistics for BMI from the GIANT consortium website (2) with SBayesR approach (3). The total number of genetic variants used in the PGS BMI calculation was 902 833. A detailed description of genotyping, quality control, and polygenic scoring is presented elsewhere (4,5). Association analysis was performed using linear mixed modelling including the family number in the models as a random factor. The models were adjusted for age and also for ten genetic principal components, when PGS was included in the model. PGS value was standardized (z-score) and the level of significance was set at $P<0.05$.

eTable 5. Characteristics in the FITSA Cohort

	n (%)	mean (SD)	n
Age (y)		68.6 (3.4)	429
BMI (kg/m^2)		27.9 (4.7)	429
Never smoker	359 (83.7)		409
Isometric HGS (N)		190.7 (57.1)	429

Note: FITSA=Finnish Twin Study on Aging, SD=Standard Deviation, BMI= Body Mass Index, HGS= Hand Grip Strength.

In the FITSA cohort, BMI did not correlate with measured HGS (β -0.369, SE 0.553, P=0.506). We did not find an association between PGS HGS and BMI (β 0.076, SE 0.242, P=0.753), and neither between PGS BMI and HGS (β -0.746, SE 3.142, P=0.812).

References:

1. Tiainen K, Sipilä S, Alen M, et al. Heritability of maximal isometric muscle strength in older female twins. *J Appl Physiol.* 2004;96(1):173-180. doi:10.1152/japplphysiol.00200.2003

2. Yengo L, Sidorenko J, Kemper KE, et al. Meta-analysis of genome-wide association studies for height and body mass index in ∼700000 individuals of European ancestry. *Hum Mol Genet.* 2018;27(20):3641-3649. doi:10.1093/hmg/ddy271

3. Lloyd-Jones LR, Zeng J, Sidorenko J, et al. Improved polygenic prediction by Bayesian multiple regression on summary statistics. *Nat Commun.* 2019;10(1):5086. doi:10.1038/s41467-019-12653-0

4. Kujala UM, Palviainen T, Pesonen P, et al. Polygenic risk scores and physical activity. *Med Sci Sports Exerc*. 2020;52(7):1518-1524. doi:10.1249/MSS.0000000000002290

5. Herranen P, Palviainen T, Rantanen T, et al. A polygenic risk score for hand grip strength predicts muscle strength and proximal and distal functional outcomes among older women. *Med Sci Sports Exerc*. 2022;54(11):1889-1896. doi:10.1249/MSS.0000000000002981

FinnGen

Full Name	Affiliation	E-mail	Role 1	Role 2
Aarno Palotie	Institute for Molecular Medicine Finland (FIMM), HiLIFE, University of Helsinki, Helsinki,	aarno.palotie@helsinki.fi	Steering Committee	Steering Committee
Mark Daly	Institute for Molecular Medicine Finland (FIMM), HiLIFE, University of Helsinki, Helsinki,	mark.daly@helsinki.fi	Steering Committee	Steering Committee
Bridget Riley-Gills	Abbvie, Chicago, IL, United States	bridget.rileygillis@abbvie.com	Steering Committee	Pharmaceutical companies
Howard Jacob	Abbvie, Chicago, IL, United States	howard.jacob@abbvie.com	Steering Committee	Pharmaceutical companies
Dirk Paul	Astra Zeneca, Cambridge, United Kingdom	dirk.paul@astrazeneca.com	Steering Committee	Pharmaceutical companies
Slavé Petrovski	Astra Zeneca, Cambridge, United Kingdom	slav.petrovski@astrazeneca.com	Steering Committee	Pharmaceutical companies
Heiko Runz	Biogen, Cambridge, MA, United States	heiko.runz@biogen.com	Steering Committee	Pharmaceutical companies
Sally John	Biogen, Cambridge, MA, United States	sally.john@biogen.com	Steering Committee	Pharmaceutical companies
George Okafo	Boehringer Ingelheim, Ingelheim am Rhein, Germany	george.okafo@boehringer-ingelheim.com	Steering Committee	Pharmaceutical companies
Nathan Lawless	Boehringer Ingelheim, Ingelheim am Rhein, Germany	nathan.lawless@boehringer-ingelheim.com	Steering Committee	Pharmaceutical companies
Heli Salminen-Mankonen	Boehringer Ingelheim, Ingelheim am Rhein, Germany	heli.salminen-mankonen@boehringer-ingel	Steering Committee	Pharmaceutical companies
Robert Plenge	Bristol Myers Squibb, New York, NY, United States	robert.plenge@bms.com	Steering Committee	Pharmaceutical companies
Joseph Maranville	Bristol Myers Squibb, New York, NY, United States	joseph.maranville@bms.com	Steering Committee	Pharmaceutical companies
Mark McCarthy	Genentech, San Francisco, CA, United States	mccarthy.mark@gene.com	Steering Committee	Pharmaceutical companies
Margaret G. Ehm	GlaxoSmithKline, Collegeville, PA, United States	meg.g.ehm@gsk.com	Steering Committee	Pharmaceutical companies
Kirsi Auro	GlaxoSmithKline, Espoo, Finland	kirsi.m.auro@gsk.com	Steering Committee	Pharmaceutical companies
Simonne Longerich	Merck, Kenilworth, NJ, United States	simonne.longerich@merck.com	Steering Committee	Pharmaceutical companies
Anders Mälarstig	Pfizer, New York, NY, United States	anders.malarstig@pfizer.com	Steering Committee	Pharmaceutical companies
Katherine Klinger	Translational Sciences, Sanofi R&D, Framingham, MA, USA	katherine.klinger@sanofi.com	Steering Committee	Pharmaceutical companies
Clement Chatelain	Translational Sciences, Sanofi R&D, Framingham, MA, USA	clement.chatelain@sanofi.com	Steering Committee	Pharmaceutical companies
Matthias Gossel	Translational Sciences, Sanofi R&D, Framingham, MA, USA	matthias.gossel@sanofi.com	Steering Committee	Pharmaceutical companies
Karol Estrada	Maze Therapeutics, San Francisco, CA, United States	kestrada@mazetx.com	Steering Committee	Pharmaceutical companies
Robert Graham	Maze Therapeutics, San Francisco, CA, United States	rgraham@mazetx.com	Steering Committee	Pharmaceutical companies
Robert Yang	Janssen Biotech, Beerse, Belgium	ryang31@its.jnj.com	Steering Committee	Pharmaceutical companies
Chris O´Donnell	Novartis Institutes for BioMedical Research, Cambridge, MA, United States	chris.odonnell@novartis.com	Steering Committee	Pharmaceutical companies
Tomi P. Mäkelä	HiLIFE, University of Helsinki, Finland, Finland	tomi.makela@helsinki.fi	Steering Committee	University of Helsinki & Biobanks
Jaakko Kaprio	Institute for Molecular Medicine Finland (FIMM), HiLIFE, University of Helsinki, Helsinki, Finland	jaakko.kaprio@helsinki.fi	Steering Committee	University of Helsinki & Biobanks
Petri Virolainen	Auria Biobank / University of Turku / Hospital District of Southwest Finland, Turku, Finland	petri.virolainen@tyks.fi	Steering Committee	University of Helsinki & Biobanks
Antti Hakanen	Auria Biobank / University of Turku / Hospital District of Southwest Finland, Turku, Finland	antti.hakanen@tyks.fi	Steering Committee	University of Helsinki & Biobanks
Terhi Kilpi	THL Biobank / Finnish Institute for Health and Welfare (THL), Helsinki, Finland	terhi.kilpi@thl.fi	Steering Committee	University of Helsinki & Biobanks
Markus Perola	THL Biobank / Finnish Institute for Health and Welfare (THL), Helsinki, Finland	markus.perola@thl.fi	Steering Committee	University of Helsinki & Biobanks
Jukka Partanen	Finnish Red Cross Blood Service / Finnish Hematology Registry and Clinical Biobank, Helsinki, Finland	jukka.partanen@veripalvelu.fi	Steering Committee	University of Helsinki & Biobanks
Anne Pitkäranta	Helsinki Biobank / Helsinki University and Hospital District of Helsinki and Uusimaa, Helsinki	anne.pitkaranta@hus.fi	Steering Committee	University of Helsinki & Biobanks
Taneli Raivio	Helsinki Biobank / Helsinki University and Hospital District of Helsinki and Uusimaa, Helsinki	taneli.raivio@hus.fi	Steering Committee	University of Helsinki & Biobanks
Jani Tikkanen	Northern Finland Biobank Borealis / University of Oulu / Northern Ostrobothnia Hospital District, Oulu, Finland	jani.tikkanen@ppshp.fi	Steering Committee	University of Helsinki & Biobanks
Raisa Serpi	Northern Finland Biobank Borealis / University of Oulu / Northern Ostrobothnia Hospital District, Oulu, Finland	raisa.serpi@ppshp.fi	Steering Committee	University of Helsinki & Biobanks
Tarja Laitinen	Finnish Clinical Biobank Tampere / University of Tampere / Pirkanmaa Hospital District, Tampere, Finland	tarja.laitinen@pshp.fi	Steering Committee	University of Helsinki & Biobanks
Veli-Matti Kosma	Biobank of Eastern Finland / University of Eastern Finland / Northern Savo Hospital District, Kuopio, Finland	veli-matti.kosma@uef.fi	Steering Committee	University of Helsinki & Biobanks
Jari Laukkanen	Central Finland Biobank / University of Jyväskylä / Central Finland Health Care District, Jyväskylä, Finland	jari.laukkanen@ksshp.fi	Steering Committee	University of Helsinki & Biobanks
Marco Hautalahti	FINBB - Finnish biobank cooperative	marco.hautalahti@finbb.fi	Steering Committee	University of Helsinki & Biobanks
Outi Tuovila	Business Finland, Helsinki, Finland	outi.tuovila@businessfinland.fi	Steering Committee	Other Experts/ Non-Voting Members
Raimo Pakkanen	Business Finland, Helsinki, Finland	raimo.pakkanen@businessfinland.fi	Steering Committee	Other Experts/ Non-Voting Members
Jeffrey Waring	Abbvie, Chicago, IL, United States	jeff.waring@abbvie.com	Scientific Committee	Pharmaceutical companies
Bridget Riley-Gillis	Abbvie, Chicago, IL, United States	bridget.rileygillis@abbvie.com	Scientific Committee	Pharmaceutical companies
Fedik Rahimov	Abbvie, Chicago, IL, United States	fedik.rahimov@abbvie.com	Scientific Committee	Pharmaceutical companies
Ioanna Tachmazidou	Astra Zeneca, Cambridge, United Kingdom	ioanna.tachmazidou@astrazeneca.com	Scientific Committee	Pharmaceutical companies
Chia-Yen Chen	Biogen, Cambridge, MA, United States	chiayen.chen@biogen.com	Scientific Committee	Pharmaceutical companies
Heiko Runz	Biogen, Cambridge, MA, United States	heiko.runz@biogen.com	Scientific Committee	Pharmaceutical companies
Zhihao Ding	Boehringer Ingelheim, Ingelheim am Rhein, Germany	zhihao.ding@boehringer-ingelheim.com	Scientific Committee	Pharmaceutical companies
Marc Jung	Boehringer Ingelheim, Ingelheim am Rhein, Germany	marc_oliver.jung@boehringer-ingelheim.cc	Scientific Committee	Pharmaceutical companies
Shameek Biswas	Bristol Myers Squibb, New York, NY, United States	Shameek.Biswas@bms.com	Scientific Committee	Pharmaceutical companies
Rion Pendergrass	Genentech, San Francisco, CA, United States	penders2@gene.com	Scientific Committee	Pharmaceutical companies
Margaret G. Ehm	GlaxoSmithKline, Collegeville, PA, United States	meg.g.ehm@gsk.com	Scientific Committee	Pharmaceutical companies
David Pulford	GlaxoSmithKline, Stevenage, United Kingdom	david.x.pulford@gsk.com	Scientific Committee	Pharmaceutical companies
Neha Raghavan	Merck, Kenilworth, NJ, United States	neha.raghavan@merck.com	Scientific Committee	Pharmaceutical companies
Adriana Huertas-Vazque	Merck, Kenilworth, NJ, United States	adriana.huertas.vazquez@merck.com	Scientific Committee	Pharmaceutical companies
Jae-Hoon Sul	Merck, Kenilworth, NJ, United States	jae.hoon.sul@merck.com	Scientific Committee	Pharmaceutical companies
Anders Mälarstig	Pfizer, New York, NY, United States	anders.malarstig@pfizer.com	Scientific Committee	Pharmaceutical companies
Xinli Hu	Pfizer, New York, NY, United States	xinli.hu@pfizer.com	Scientific Committee	Pharmaceutical companies
Åsa Hedman	Pfizer, New York, NY, United States	asa.hedman@pfizer.com	Scientific Committee	Pharmaceutical companies
Katherine Klinger	Translational Sciences, Sanofi R&D, Framingham, MA, USA	katherine.klinger@sanofi.com	Scientific Committee	Pharmaceutical companies
Robert Graham	Maze Therapeutics, San Francisco, CA, United States	rgraham@mazetx.com	Scientific Committee	Pharmaceutical companies
Manuel Rivas	Maze Therapeutics, San Francisco, CA, United States	mrivas@mazetx.com	Scientific Committee	Pharmaceutical companies
Dawn Waterworth	Janssen Research & Development, LLC, Spring House, PA, United States	dwaterwo@its.jnj.com	Scientific Committee	Pharmaceutical companies
Nicole Renaud	Novartis Institutes for BioMedical Research, Cambridge, MA, United States	nicole.renaud@novartis.com	Scientific Committee	Pharmaceutical companies
Ma´en Obeidat	Novartis Institutes for BioMedical Research, Cambridge, MA, United States	maen.obeidat@novartis.com	Scientific Committee	Pharmaceutical companies
Samuli Ripatti	Institute for Molecular Medicine Finland (FIMM), HiLIFE, University of Helsinki, Helsinl	samuli.ripatti@helsinki.fi	Scientific Committee	University of Helsinki & Biobanks
Johanna Schleutker	Auria Biobank / Univ. of Turku / Hospital District of Southwest Finland, Turku, Finland	johanna.schleutker@utu.fi	Scientific Committee	University of Helsinki & Biobanks
Markus Perola	THL Biobank / Finnish Institute for Health and Welfare (THL), Helsinki, Finland	markus.perola@thl.fi	Scientific Committee	University of Helsinki & Biobanks
Mikko Arvas	Finnish Red Cross Blood Service / Finnish Hematology Registry and Clinical Biobank, Helsinki, Finland	mikko.arvas@veripalvelu.fi	Scientific Committee	University of Helsinki & Biobanks
Olli Carpén	Helsinki Biobank / Helsinki University and Hospital District of Helsinki and Uusimaa, Helsinki	olli.carpen@helsinki.fi	Scientific Committee	University of Helsinki & Biobanks
Reetta Hinttala	Northern Finland Biobank Borealis / University of Oulu / Northern Ostrobothnia Hospital District, Oulu, Finland	reetta.hinttala@oulu.fi	Scientific Committee	University of Helsinki & Biobanks
Johannes Kettunen	Northern Finland Biobank Borealis / University of Oulu / Northern Ostrobothnia Hospital District, Oulu, Finland	johannes.kettunen@oulu.fi	Scientific Committee	University of Helsinki & Biobanks
Arto Mannermaa	Biobank of Eastern Finland / University of Eastern Finland / Northern Savo Hospital District, Kuopio, Finland	arto.mannermaa@uef.fi	Scientific Committee	University of Helsinki & Biobanks
Katriina Aalto-Setälä	Faculty of Medicine and Health Technology, Tampere University, Tampere, Finland	katriina.aalto-setala@tuni.fi	Scientific Committee	University of Helsinki & Biobanks
Mika Kähönen	Finnish Clinical Biobank Tampere / University of Tampere / Pirkanmaa Hospital District, Tampere, Finland	mika.kahonen@uta.fi	Scientific Committee	University of Helsinki & Biobanks
Jari Laukkanen	Central Finland Biobank / University of Jyväskylä / Central Finland Health Care District, Jyväskylä, Finland	jari.laukkanen@ksshp.fi	Scientific Committee	University of Helsinki & Biobanks
Johanna Mäkelä	FINBB - Finnish biobank cooperative	johanna.makela@finbb.fi	Scientific Committee	University of Helsinki & Biobanks
Reetta Kälviäinen	Northern Savo Hospital District, Kuopio, Finland	reetta.kalviainen@kuh.fi	Clinical Groups	Neurology Group
Valtteri Julkunen	Northern Savo Hospital District, Kuopio, Finland	valtteri.julkunen@kuh.fi	Clinical Groups	Neurology Group
Hilkka Soininen	Northern Savo Hospital District, Kuopio, Finland	hilkka.soininen@uef.fi	Clinical Groups	Neurology Group
Anne Remes	Northern Ostrobothnia Hospital District, Oulu, Finland	anne.remes@oulu.fi	Clinical Groups	Neurology Group
Mikko Hiltunen	University of Eastern Finland, Kuopio, Finland	mikko.hiltunen@uef.fi	Clinical Groups	Neurology Group
Jukka Peltola	Pirkanmaa Hospital District, Tampere, Finland	jukka.peltola@pshp.fi	Clinical Groups	Neurology Group
Minna Raivio	Hospital District of Helsinki and Uusimaa, Helsinki, Finland	minna.raivio@geri.fi	Clinical Groups	Neurology Group
Pentti Tienari	Hospital District of Helsinki and Uusimaa, Helsinki, Finland	pentti.tienari@hus.fi	Clinical Groups	Neurology Group
Juha Rinne	Hospital District of Southwest Finland, Turku, Finland	juha.rinne@tyks.fi	Clinical Groups	Neurology Group
Roosa Kallionpää	Hospital District of Southwest Finland, Turku, Finland	roosa.kallionpaa@tyks.fi	Clinical Groups	Neurology Group
Juulia Partanen	Institute for Molecular Medicine Finland, HiLIFE, University of Helsinki, Finland	juulia.partanen@helsinki.fi	Clinical Groups	Neurology Group
Ali Abbasi	Abbvie, Chicago, IL, United States	ali.abbasi@abbvie.com	Clinical Groups	Neurology Group
Adam Ziemann	Abbvie, Chicago, IL, United States	adam.ziemann@abbvie.com	Clinical Groups	Neurology Group
Nizar Smaoui	Abbvie, Chicago, IL, United States	nizar.smaoui@abbvie.com	Clinical Groups	Neurology Group
Anne Lehtonen	Abbvie, Chicago, IL, United States	anne.lehtonen@abbvie.com	Clinical Groups	Neurology Group
Susan Eaton	Biogen, Cambridge, MA, United States	susan.eaton@biogen.com	Clinical Groups	Neurology Group
Heiko Runz	Biogen, Cambridge, MA, United States	heiko.runz@biogen.com	Clinical Groups	Neurology Group
Sanni Lahdenperä	Biogen, Cambridge, MA, United States	sanni.lahdenpera@biogen.com	Clinical Groups	Neurology Group

Shameek Biswas	Bristol Myers Squibb, New York, NY, United States	shameek.biswas@bms.com	Clinical Groups	**Neurology Group**
Natalie Bowers	Genentech, San Francisco, CA, United States	bowersn1@gene.com	Clinical Groups	**Neurology Group**
Edmond Teng	Genentech, San Francisco, CA, United States	teng.edmond@gene.com	Clinical Groups	**Neurology Group**
Rion Pendergrass	Genentech, San Francisco, CA, United States	penders2@gene.com	Clinical Groups	**Neurology Group**
Fanli Xu	GlaxoSmithKline, Brentford, United Kingdom	chun-fang.2.xu@gsk.com	Clinical Groups	**Neurology Group**
David Pulford	GlaxoSmithKline, Stevenage, United Kingdom	david.x.pulford@gsk.com	Clinical Groups	**Neurology Group**
Kirsi Auro	GlaxoSmithKline, Espoo, Finland	kirsi.m.auro@gsk.com	Clinical Groups	**Neurology Group**
Laura Addis	GlaxoSmithKline, Brentford, United Kingdom	laura.x.addis@gsk.com	Clinical Groups	**Neurology Group**
John Eicher	GlaxoSmithKline, Brentford, United Kingdom	john.d.eicher@gsk.com	Clinical Groups	**Neurology Group**
Qingqin S Li	Janssen Research & Development, LLC, Titusville, NJ 08560, United States	QLi2@its.jnj.com	Clinical Groups	**Neurology Group**
Karen He	Janssen Research & Development, LLC, Spring House, PA, United States	khe2@its.jnj.com	Clinical Groups	**Neurology Group**
Ekaterina Khramtsova	Janssen Research & Development, LLC, Spring House, PA, United States	ekhramts@its.jnj.com	Clinical Groups	**Neurology Group**
Neha Raghavan	Merck, Kenilworth, NJ, United States	neha.raghavan@merck.com	Clinical Groups	**Neurology Group**
Martti Färkkilä	Hospital District of Helsinki and Uusimaa, Helsinki, Finland	martti.farkkila@hus.fi	Clinical Groups	**Gastroenterology Group**
Jukka Koskela	Hospital District of Helsinki and Uusimaa, Helsinki, Finland	jukka.koskela@helsinki.fi	Clinical Groups	**Gastroenterology Group**
Sampsa Pikkarainen	Hospital District of Helsinki and Uusimaa, Helsinki, Finland	sampsa.pikkarainen@hus.fi	Clinical Groups	**Gastroenterology Group**
Airi Jussila	Pirkanmaa Hospital District, Tampere, Finland	airi.jussila@pshp.fi	Clinical Groups	**Gastroenterology Group**
Katri Kaukinen	Pirkanmaa Hospital District, Tampere, Finland	katri.kaukinen@tuni.fi	Clinical Groups	**Gastroenterology Group**
Timo Blomster	Northern Ostrobothnia Hospital District, Oulu, Finland	timo.blomster@ppshp.fi	Clinical Groups	**Gastroenterology Group**
Mikko Kiviniemi	Northern Savo Hospital District, Kuopio, Finland	mikko.kiviniemi@kuh.fi	Clinical Groups	**Gastroenterology Group**
Markku Voutilainen	Hospital District of Southwest Finland, Turku, Finland	markku.voutilainen@tyks.fi	Clinical Groups	**Gastroenterology Group**
Mark Daly	Institute for Molecular Medicine, Finland (FIMM), HiLIFE, University of Helsinki, Helsinki, Finland; Broad Institute of MIT and Harvard; Massachusetts General Hospital	mark.daly@helsinki.fi	Clinical Groups	**Gastroenterology Group**
Ali Abbasi	Abbvie, Chicago, IL, United States	ali.abbasi@abbvie.com	Clinical Groups	**Gastroenterology Group**
Jeffrey Waring	Abbvie, Chicago, IL, United States	jeff.waring@abbvie.com	Clinical Groups	**Gastroenterology Group**
Nizar Smaoui	Abbvie, Chicago, IL, United States	nizar.smaoui@abbvie.com	Clinical Groups	**Gastroenterology Group**
Fedik Rahimov	Abbvie, Chicago, IL, United States	fedik.rahimov@abbvie.com	Clinical Groups	**Gastroenterology Group**
Anne Lehtonen	Abbvie, Chicago, IL, United States	anne.lehtonen@abbvie.com	Clinical Groups	**Gastroenterology Group**
Tim Lu	Genentech, San Francisco, CA, United States	lut8@gene.com	Clinical Groups	**Gastroenterology Group**
Natalie Bowers	Genentech, San Francisco, CA, United States	bowersn1@gene.com	Clinical Groups	**Gastroenterology Group**
Rion Pendergrass	Genentech, San Francisco, CA, United States	penders2@gene.com	Clinical Groups	**Gastroenterology Group**
Linda McCarthy	GlaxoSmithKline, Brentford, United Kingdom	linda.c.mccarthy@gsk.com	Clinical Groups	**Gastroenterology Group**
Amy Hart	Janssen Research & Development, LLC, Spring House, PA, United States	ahart13@its.jnj.com	Clinical Groups	**Gastroenterology Group**
Meijian Guan	Janssen Research & Development, LLC, Spring House, PA, United States	mguan4@its.jnj.com	Clinical Groups	**Gastroenterology Group**
Jason Miller	Merck, Kenilworth, NJ, United States	jason.miller4@merck.com	Clinical Groups	**Gastroenterology Group**
Kirsi Kalpala	Pfizer, New York, NY, United States	kirsi.kalpala@pfizer.com	Clinical Groups	**Gastroenterology Group**
Melissa Miller	Pfizer, New York, NY, United States	melissa.r.miller@pfizer.com	Clinical Groups	**Gastroenterology Group**
Xinli Hu	Pfizer, New York, NY, United States	xinli.hu@pfizer.com	Clinical Groups	**Gastroenterology Group**
Kari Eklund	Hospital District of Helsinki and Uusimaa, Helsinki, Finland	kari.eklund@hus.fi	Clinical Groups	**Rheumatology Group**
Antti Palomäki	Hospital District of Southwest Finland, Turku, Finland	ajpalo@utu.fi	Clinical Groups	**Rheumatology Group**
Pia Isomäki	Pirkanmaa Hospital District, Tampere, Finland	pia.isomaki@pshp.fi	Clinical Groups	**Rheumatology Group**
Laura Pirilä	Hospital District of Southwest Finland, Turku, Finland	laura.pirila@fimnet.fi,laura.pirila@tyks.fi	Clinical Groups	**Rheumatology Group**
Oili Kaipiainen-Seppänen	Northern Savo Hospital District, Kuopio, Finland	oili.kaipiainen-seppanen@kuh.fi	Clinical Groups	**Rheumatology Group**
Johanna Huhtakangas	Northern Ostrobothnia Hospital District, Oulu, Finland	johanna.huhtakangas@kuh.fi	Clinical Groups	**Rheumatology Group**
Nina Mars	Institute for Molecular Medicine Finland (FIMM), HiLIFE, University of Helsinki, Helsink	nina.mars@helsinki.fi	Clinical Groups	**Rheumatology Group**
Ali Abbasi	Abbvie, Chicago, IL, United States	ali.abbasi@abbvie.com	Clinical Groups	**Rheumatology Group**
Jeffrey Waring	Abbvie, Chicago, IL, United States	jeff.waring@abbvie.com	Clinical Groups	**Rheumatology Group**
Fedik Rahimov	Abbvie, Chicago, IL, United States	fedik.rahimov@abbvie.com	Clinical Groups	**Rheumatology Group**
Apinya Lertratanakul	Abbvie, Chicago, IL, United States	apinya.lertratanakul@abbvie.com	Clinical Groups	**Rheumatology Group**
Nizar Smaoui	Abbvie, Chicago, IL, United States	nizar.smaoui@abbvie.com	Clinical Groups	**Rheumatology Group**
Anne Lehtonen	Abbvie, Chicago, IL, United States	anne.lehtonen@abbvie.com	Clinical Groups	**Rheumatology Group**
Coralie Viollet	AstraZeneca, Cambridge, United Kingdom	coralie.viollet@astrazeneca.com	Clinical Groups	**Rheumatology Group**
Marla Hochfeld	Bristol Myers Squibb, New York, NY, United States	mhochfeld@celgene.com	Clinical Groups	**Rheumatology Group**
Natalie Bowers	Genentech, San Francisco, CA, United States	bowersn1@gene.com	Clinical Groups	**Rheumatology Group**
Rion Pendergrass	Genentech, San Francisco, CA, United States	penders2@gene.com	Clinical Groups	**Rheumatology Group**
Jorge Esparza Gordillo	GlaxoSmithKline, Brentford, United Kingdom	jorge.x.esparza-gordillo@gsk.com	Clinical Groups	**Rheumatology Group**
Kirsi Auro	GlaxoSmithKline, Espoo, Finland	kirsi.m.auro@gsk.com	Clinical Groups	**Rheumatology Group**
Dawn Waterworth	Janssen Research & Development, LLC, Spring House, PA, United States	dwaterwo@its.jnj.com	Clinical Groups	**Rheumatology Group**
Fabiana Farias	Merck, Kenilworth, NJ, United States	fabiana.farias@merck.com	Clinical Groups	**Rheumatology Group**
Kirsi Kalpala	Pfizer, New York, NY, United States	kirsi.kalpala@pfizer.com	Clinical Groups	**Rheumatology Group**
Nan Bing	Pfizer, New York, NY, United States	nan.bing@pfizer.com	Clinical Groups	**Rheumatology Group**
Xinli Hu	Pfizer, New York, NY, United States	xinli.hu@pfizer.com	Clinical Groups	**Rheumatology Group**
Tarja Laitinen	Pirkanmaa Hospital District, Tampere, Finland	tarja.laitinen@pshp.fi	Clinical Groups	**Pulmonology Group**
Margit Pelkonen	Northern Savo Hospital District, Kuopio, Finland	margit.pelkonen@kuh.fi	Clinical Groups	**Pulmonology Group**
Paula Kauppi	Hospital District of Helsinki and Usimaa, Helsinki, Finland	paula.kauppi@hus.fi	Clinical Groups	**Pulmonology Group**
Hannu Kankaanranta	University of Gothenburg, Gothenburg, Sweden/ Seinäjoki Central Hospital, Seinäjoki, Finland/ Tampere University, Tampere, Finland	hannu.kankaanranta@tuni.fi	Clinical Groups	**Pulmonology Group**
Terttu Harju	Northern Ostrobothnia Hospital District, Oulu, Finland	terttu.harju@oulu.fi	Clinical Groups	**Pulmonology Group**
Riitta Lahesmaa	Hospital District of Southwest Finland, Turku, Finland	rilahes@utu.fi	Clinical Groups	**Pulmonology Group**
Nizar Smaoui	Abbvie, Chicago, IL, United States	nizar.smaoui@abbvie.com	Clinical Groups	**Pulmonology Group**
Coralie Viollet	AstraZeneca, Cambridge, United Kingdom	coralie.viollet@astrazeneca.com	Clinical Groups	**Pulmonology Group**
Susan Eaton	Biogen, Cambridge, MA, United States	susan.eaton@biogen.com	Clinical Groups	**Pulmonology Group**
Hubert Chen	Genentech, San Francisco, CA, United States	chenh37@gene.com	Clinical Groups	**Pulmonology Group**
Rion Pendergrass	Genentech, San Francisco, CA, United States	penders2@gene.com	Clinical Groups	**Pulmonology Group**
Natalie Bowers	Genentech, San Francisco, CA, United States	bowersn1@gene.com	Clinical Groups	**Pulmonology Group**
Joanna Betts	GlaxoSmithKline, Brentford, United Kingdom	joanna.c.betts@gsk.com	Clinical Groups	**Pulmonology Group**
Kirsi Auro	GlaxoSmithKline, Espoo, Finland	kirsi.m.auro@gsk.com	Clinical Groups	**Pulmonology Group**
Rajashree Mishra	GlaxoSmithKline, Brentford, United Kingdom	rajashree.x.mishra@gsk.com	Clinical Groups	**Pulmonology Group**
Majd Mouded	Novartis, Basel, Switzerland	majd.mouded@novartis.com	Clinical Groups	**Pulmonology Group**
Debby Ngo	Novartis, Basel, Switzerland	debby.ngo@novartis.com	Clinical Groups	**Pulmonology Group**
Teemu Niiranen	Finnish Institute for Health and Welfare (THL), Helsinki, Finland	teemu.niiranen@thl.fi	Clinical Groups	**Cardiometabolic Diseases Group**
Felix Vaura	Finnish Institute for Health and Welfare (THL), Helsinki, Finland	fechva@utu.fi	Clinical Groups	**Cardiometabolic Diseases Group**
Veikko Salomaa	Finnish Institute for Health and Welfare (THL), Helsinki, Finland	veikko.salomaa@thl.fi	Clinical Groups	**Cardiometabolic Diseases Group**
Kaj Metsärinne	Hospital District of Southwest Finland, Turku, Finland	kaj.metsarinne@tyks.fi	Clinical Groups	**Cardiometabolic Diseases Group**
Jenni Aittokallio	Hospital District of Southwest Finland, Turku, Finland	jemato@utu.fi	Clinical Groups	**Cardiometabolic Diseases Group**
Mika Kähönen	Pirkanmaa Hospital District, Tampere, Finland	mika.kahonen@uta.fi	Clinical Groups	**Cardiometabolic Diseases Group**
Jussi Hernesniemi	Pirkanmaa Hospital District, Tampere, Finland	jussi.hernesniemi@tuni.fi	Clinical Groups	**Cardiometabolic Diseases Group**
Daniel Gordin	Hospital District of Helsinki and Uusimaa, Helsinki, Finland	daniel.gordin@hus.fi	Clinical Groups	**Cardiometabolic Diseases Group**
Juha Sinisalo	Hospital District of Helsinki and Uusimaa, Helsinki, Finland	juha.sinisalo@hus.fi	Clinical Groups	**Cardiometabolic Diseases Group**
Marja-Riitta Taskinen	Hospital District of Helsinki and Uusimaa, Helsinki, Finland	marja-riitta.taskinen@helsinki.fi	Clinical Groups	**Cardiometabolic Diseases Group**
Tiinamaija Tuomi	Hospital District of Helsinki and Uusimaa, Helsinki, Finland	tiinamaija.tuomi@hus.fi	Clinical Groups	**Cardiometabolic Diseases Group**
Timo Hiltunen	Hospital District of Helsinki and Uusimaa, Helsinki, Finland	timo.hiltunen@hus.fi	Clinical Groups	**Cardiometabolic Diseases Group**
Jari Laukkanen	Central Finland Health Care District, Jyväskylä, Finland	jari.laukkanen@ksshp.fi	Clinical Groups	**Cardiometabolic Diseases Group**
Amanda Elliott	Institute for Molecular Medicine Finland (FIMM), HiLIFE, University of Helsinki, Helsinki, Finland; Broad Institute, Cambridge, MA, USA and Massachusetts General Hospital, Boston, MA, USA	aelliott@broadinstitute.org	Clinical Groups	**Cardiometabolic Diseases Group**
Mary Pat Reeve	Institute for Molecular Medicine Finland (FIMM), HiLIFE, University of Helsinki, Helsink	mary.reeve@helsinki.fi	Clinical Groups	**Cardiometabolic Diseases Group**
Sanni Ruotsalainen	Institute for Molecular Medicine Finland (FIMM), HiLIFE, University of Helsinki, Helsink	sanni.ruotsalainen@helsinki.fi	Clinical Groups	**Cardiometabolic Diseases Group**
Dirk Paul	Astra Zeneca, Cambridge, United Kingdom	dirk.paul@astrazeneca.com	Clinical Groups	**Cardiometabolic Diseases Group**
Natalie Bowers	Genentech, San Francisco, CA, United States	bowersn1@gene.com	Clinical Groups	**Cardiometabolic Diseases Group**
Rion Pendergrass	Genentech, San Francisco, CA, United States	penders2@gene.com	Clinical Groups	**Cardiometabolic Diseases Group**
Audrey Chu	GlaxoSmithKline, Brentford, United Kingdom	audrey.y.chu@gsk.com	Clinical Groups	**Cardiometabolic Diseases Group**
Kirsi Auro	GlaxoSmithKline, Espoo, Finland	kirsi.m.auro@gsk.com	Clinical Groups	**Cardiometabolic Diseases Group**
Dermot Reilly	Janssen Research & Development, LLC, Boston, MA, United States	dreill11@its.jnj.com	Clinical Groups	**Cardiometabolic Diseases Group**
Mike Mendelson	Novartis, Boston, MA, United States	mike.mendelson@novartis.com	Clinical Groups	**Cardiometabolic Diseases Group**
Jaakko Parkkinen	Pfizer, New York, NY, United States	jaakko.parkkinen@pfizer.com	Clinical Groups	**Cardiometabolic Diseases Group**
Melissa Miller	Pfizer, New York, NY, United States	melissa.r.miller@pfizer.com	Clinical Groups	**Cardiometabolic Diseases Group**
Tuomo Meretoja	Hospital District of Helsinki and Uusimaa, Helsinki, Finland	tuomo.meretoja@hus.fi	Clinical Groups	**Oncology Group**
Heikki Joensuu	Hospital District of Helsinki and Uusimaa, Helsinki, Finland	heikki.joensuu@hus.fi	Clinical Groups	**Oncology Group**
Olli Carpén	Hospital District of Helsinki and Uusimaa, Helsinki, Finland	olli.carpen@helsinki.fi	Clinical Groups	**Oncology Group**
Johanna Mattson	Hospital District of Helsinki and Uusimaa, Helsinki, Finland	johanna.mattson@hus.fi	Clinical Groups	**Oncology Group**
Eveliina Salminen	Hospital District of Helsinki and Uusimaa, Helsinki, Finland	eveliina.e.salminen@hus.fi	Clinical Groups	**Oncology Group**
Annika Auranen	Pirkanmaa Hospital District , Tampere, Finland	anaura@utu.fi	Clinical Groups	**Oncology Group**
Peeter Karihtala	Northern Ostrobothnia Hospital District, Oulu, Finland	peeter.karihtala@oulu.fi	Clinical Groups	**Oncology Group**
Päivi Auvinen	Northern Savo Hospital District, Kuopio, Finland	paivi.auvinen@kuh.fi	Clinical Groups	**Oncology Group**
Klaus Elenius	Hospital District of Southwest Finland, Turku, Finland	klaus.elenius@utu.fi	Clinical Groups	**Oncology Group**
Johanna Schleutker	Hospital District of Southwest Finland, Turku, Finland	johanna.schleutker@utu.fi	Clinical Groups	**Oncology Group**
Esa Pitkänen	Institute for Molecular Medicine Finland (FIMM), HiLIFE, University of Helsinki, Helsink	esa.pitkanen@helsinki.fi	Clinical Groups	**Oncology Group**
Nina Mars	Institute for Molecular Medicine Finland (FIMM), HiLIFE, University of Helsinki, Helsink	nina.mars@helsinki.fi	Clinical Groups	**Oncology Group**

Mark Daly	Institute for Molecular Medicine Finland (FIMM), HiLIFE, University of Helsinki, Helsinki, Finland; Broad Institute of MIT and Harvard; Massachusetts General Hospital	mark.daly@helsinki.fi	Clinical Groups	Oncology Group
Relja Popovic	Abbvie, Chicago, IL, United States	relja.popovic@abbvie.com	Clinical Groups	Oncology Group
Jeffrey Waring	Abbvie, Chicago, IL, United States	jeff.waring@abbvie.com	Clinical Groups	Oncology Group
Bridget Riley-Gillis	Abbvie, Chicago, IL, United States	bridget.rileygillis@abbvie.com	Clinical Groups	Oncology Group
Anne Lehtonen	Abbvie, Chicago, IL, United States	anne.lehtonen@abbvie.com	Clinical Groups	Oncology Group
Margarete Fabre	AstraZeneca, Cambridge, United Kingdom	margarete.fabre@astrazeneca.com	Clinical Groups	Oncology Group
Jennifer Schutzman	Genentech, San Francisco, CA, United States	schutzman.jennifer@gene.com	Clinical Groups	Oncology Group
Natalie Bowers	Genentech, San Francisco, CA, United States	bowersn1@gene.com	Clinical Groups	Oncology Group
Rion Pendergrass	Genentech, San Francisco, CA, United States	penders2@gene.com	Clinical Groups	Oncology Group
Diptee Kulkarni	GlaxoSmithKline, Brentford, United Kingdom	diptee.a.kulkarni@gsk.com	Clinical Groups	Oncology Group
Kirsi Auro	GlaxoSmithKline, Espoo, Finland	kirsi.m.auro@gsk.com	Clinical Groups	Oncology Group
Alessandro Porello	Janssen Research & Development, LLC, Spring House, PA, United States	APorrell@ITS.JNJ.com	Clinical Groups	Oncology Group
Andrey Loboda	Merck, Kenilworth, NJ, United States	andrey_loboda@merck.com	Clinical Groups	Oncology Group
Heli Lehtonen	Pfizer, New York, NY, United States	heli.lehtonen@pfizer.com	Clinical Groups	Oncology Group
Stefan McDonough	Pfizer, New York, NY, United States	stefan.McDonough@pfizer.com	Clinical Groups	Oncology Group
Sauli Vuoti	Janssen-Cilag Oy, Espoo, Finland	svuoti@its.jnj.com	Clinical Groups	Oncology Group
Kai Kaarniranta	Northern Savo Hospital District, Kuopio, Finland; Department of Molecular Genetics, University of Lodz, Lodz, Poland	kai.kaarniranta@uef.fi	Clinical Groups	Opthalmology Group
Joni A Turunen	Helsinki University Hospital and University of Helsinki, Helsinki, Finland; Eye Genetics Group, Folkhälsan Research Center, Helsinki, Finland	joni.turunen@helsinki.fi	Clinical Groups	Opthalmology Group
Terhi Ollila	Hospital District of Helsinki and Uusimaa, Helsinki, Finland	terhi.ollila@hus.fi	Clinical Groups	Opthalmology Group
Hannu Uusitalo	Pirkanmaa Hospital District, Tampere, Finland	hannu.uusitalo@tuni.fi	Clinical Groups	Opthalmology Group
Juha Karjalainen	Institute for Molecular Medicine Finland (FIMM), HiLIFE, University of Helsinki, Helsink	juha.karjalainen@helsinki.fi	Clinical Groups	Opthalmology Group
Esa Pitkänen	Institute for Molecular Medicine Finland (FIMM), HiLIFE, University of Helsinki, Helsink	esa.pitkanen@helsinki.fi	Clinical Groups	Opthalmology Group
Mengzhen Liu	Abbvie, Chicago, IL, United States	mengzhen.liu@abbvie.com	Clinical Groups	Opthalmology Group
Heiko Runz	Biogen, Cambridge, MA, United States	heiko.runz@biogen.com	Clinical Groups	Opthalmology Group
Stephanie Loomis	Biogen, Cambridge, MA, United States	stephanie.loomis@biogen.com	Clinical Groups	Opthalmology Group
Erich Strauss	Genentech, San Francisco, CA, United States	strauss.erich@gene.com	Clinical Groups	Opthalmology Group
Natalie Bowers	Genentech, San Francisco, CA, United States	bowersn1@gene.com	Clinical Groups	Opthalmology Group
Hao Chen	Genentech, San Francisco, CA, United States	haoc@gene.com	Clinical Groups	Opthalmology Group
Rion Pendergrass	Genentech, San Francisco, CA, United States	penders2@gene.com	Clinical Groups	Opthalmology Group
Kaisa Tasanen	Northern Ostrobothnia Hospital District, Oulu, Finland	kaisa.tasanen-maatta@oulu.fi	Clinical Groups	Dermatology Group
Laura Huilaja	Northern Ostrobothnia Hospital District, Oulu, Finland	laura.huilaja@oulu.fi	Clinical Groups	Dermatology Group
Katariina Hannula-Joupp	Hospital District of Helsinki and Uusimaa, Helsinki, Finland	katariina.hannula-jouppi@hus.fi	Clinical Groups	Dermatology Group
Teea Salmi	Pirkanmaa Hospital District, Tampere, Finland	teea.salmi@pshp.fi	Clinical Groups	Dermatology Group
Sirkku Peltonen	Hospital District of Southwest Finland, Turku, Finland	sipelto@utu.fi	Clinical Groups	Dermatology Group
Leena Koulu	Hospital District of Southwest Finland, Turku, Finland	leena.koulu@tyks.fi	Clinical Groups	Dermatology Group
Nizar Smaoui	Abbvie, Chicago, IL, United States	nizar.smaoui@abbvie.com	Clinical Groups	Dermatology Group
Fedik Rahimov	Abbvie, Chicago, IL, United States	fedik.rahimov@abbvie.com	Clinical Groups	Dermatology Group
Anne Lehtonen	Abbvie, Chicago, IL, United States	anne.lehtonen@abbvie.com	Clinical Groups	Dermatology Group
David Choy	Genentech, San Francisco, CA, United States	choy.david@gene.com	Clinical Groups	Dermatology Group
Rion Pendergrass	Genentech, San Francisco, CA, United States	penders2@gene.com	Clinical Groups	Dermatology Group
Dawn Waterworth	Janssen Research & Development, LLC, Spring House, PA, United States	dwaterwo@its.jnj.com	Clinical Groups	Dermatology Group
Kirsi Kalpala	Pfizer, New York, NY, United States	kirsi.kalpala@pfizer.com	Clinical Groups	Dermatology Group
Ying Wu	Pfizer, New York, NY, United States	ying.wu3@pfizer.com	Clinical Groups	Dermatology Group
Pirkko Pussinen	Hospital District of Helsinki and Uusimaa, Helsinki, Finland	pirkko.pussinen@helsinki.fi	Clinical Groups	Odontology Group
Aino Salminen	Hospital District of Helsinki and Uusimaa, Helsinki, Finland	aino.m.salminen@helsinki.fi	Clinical Groups	Odontology Group
Tuula Salo	Hospital District of Helsinki and Uusimaa, Helsinki, Finland	tuula.salo@helsinki.fi	Clinical Groups	Odontology Group
David Rice	Hospital District of Helsinki and Uusimaa, Helsinki, Finland	david.rice@helsinki.fi	Clinical Groups	Odontology Group
Pekka Nieminen	Hospital District of Helsinki and Uusimaa, Helsinki, Finland	pekka.nieminen@helsinki.fi	Clinical Groups	Odontology Group
Ulla Palotie	Hospital District of Helsinki and Uusimaa, Helsinki, Finland	ulla.palotie@helsinki.fi	Clinical Groups	Odontology Group
Maria Siponen	Northern Savo Hospital District, Kuopio, Finland	maria.siponen@uef.fi	Clinical Groups	Odontology Group
Liisa Suominen	Northern Savo Hospital District, Kuopio, Finland	liisa.suominen@uef.fi	Clinical Groups	Odontology Group
Päivi Mäntylä	Northern Savo Hospital District, Kuopio, Finland	paivi.mantyla@uef.fi	Clinical Groups	Odontology Group
Ulvi Gursoy	Hospital District of Southwest Finland, Turku, Finland	ulvi.gursoy@utu.fi	Clinical Groups	Odontology Group
Vuokko Anttonen	Northern Ostrobothnia Hospital District, Oulu, Finland	vuokko.anttonen@oulu.fi	Clinical Groups	Odontology Group
Kirsi Sipilä	Research Unit of Oral Health Sciences Faculty of Medicine, University of Oulu, Oulu, Finland; Medical Research Center, Oulu, Oulu University Hospital and University of Oulu, Oulu, Finland	kirsi.sipila@oulu.fi	Clinical Groups	Odontology Group
Rion Pendergrass	Genentech, San Francisco, CA, United States	pendergrass.sarah@gene.com	Clinical Groups	Odontology Group
Hannele Laivuori	Institute for Molecular Medicine Finland (FIMM), HiLIFE, University of Helsinki, Helsink	hannele.laivuori@helsinki.fi	Clinical Groups	Women's Health and Reproduction Group
Venla Kurra	Pirkanmaa Hospital District, Tampere, Finland	venla.kurra@tuni.fi	Clinical Groups	Women's Health and Reproduction Group
Laura Kotaniemi-Taloner	Pirkanmaa Hospital District, Tampere, Finland	laura.kotaniemi-talonen@tuni.fi	Clinical Groups	Women's Health and Reproduction Group
Oskari Heikinheimo	Hospital District of Helsinki and Uusimaa, Helsinki, Finland	oskari.heikinheimo@helsinki.fi	Clinical Groups	Women's Health and Reproduction Group
Ilkka Kalliala	Hospital District of Helsinki and Uusimaa, Helsinki, Finland	ilkka.kalliala@hus.fi	Clinical Groups	Women's Health and Reproduction Group
Lauri Aaltonen	Hospital District of Helsinki and Uusimaa, Helsinki, Finland	lauri.aaltonen@helsinki.fi	Clinical Groups	Women's Health and Reproduction Group
Varpu Jokimaa	Hospital District of Southwest Finland, Turku, Finland	varpu.jokimaa@utu.fi	Clinical Groups	Women's Health and Reproduction Group
Johannes Kettunen	Northern Ostrobothnia Hospital District, Oulu, Finland	Johannes.Kettunen@oulu.fi	Clinical Groups	Women's Health and Reproduction Group
Marja Vääräsmäki	Northern Ostrobothnia Hospital District, Oulu, Finland	marja.vaarasmaki@oulu.fi	Clinical Groups	Women's Health and Reproduction Group
Outi Uimari	Northern Ostrobothnia Hospital District, Oulu, Finland	outi.uimari@oulu.fi	Clinical Groups	Women's Health and Reproduction Group
Laure Morin-Papunen	Northern Ostrobothnia Hospital District, Oulu, Finland	lmp@cc.oulu.fi	Clinical Groups	Women's Health and Reproduction Group
Maarit Niinimäki	Northern Ostrobothnia Hospital District, Oulu, Finland	maarit.niinimaki@oulu.fi	Clinical Groups	Women's Health and Reproduction Group
Terhi Piltonen	Northern Ostrobothnia Hospital District, Oulu, Finland	terhi.piltonen@oulu.fi	Clinical Groups	Women's Health and Reproduction Group
Katja Kivinen	Institute for Molecular Medicine Finland (FIMM), HiLIFE, University of Helsinki, Helsink	katja.kivinen@helsinki.fi	Clinical Groups	Women's Health and Reproduction Group
Elisabeth Widen	Institute for Molecular Medicine Finland (FIMM), HiLIFE, University of Helsinki, Helsink	elisabeth.widen@helsinki.fi	Clinical Groups	Women's Health and Reproduction Group
Taru Tukiainen	Institute for Molecular Medicine Finland (FIMM), HiLIFE, University of Helsinki, Helsink	taru.tukiainen@helsinki.fi	Clinical Groups	Women's Health and Reproduction Group
Mary Pat Reeve	Institute for Molecular Medicine Finland (FIMM), HiLIFE, University of Helsinki, Helsink	mary.reeve@helsinki.fi	Clinical Groups	Women's Health and Reproduction Group
Mark Daly	Institute for Molecular Medicine Finland (FIMM), HiLIFE, University of Helsinki, Helsinki, Finland; Broad Institute of MIT and Harvard; Massachusetts General Hospital	mark.daly@helsinki.fi	Clinical Groups	Women's Health and Reproduction Group
Niko Välimäki	University of Helsinki, Helsinki, Finland	niko.valimaki@helsinki.fi	Clinical Groups	Women's Health and Reproduction Group
Eija Laakkonen	University of Jyväskylä, Jyväskylä, Finland	eija.k.laakkonen@jyu.fi	Clinical Groups	Women's Health and Reproduction Group
Jaakko Tyrmi	University of Oulu, Oulu, Finland / University of Tampere, Tampere, Finland	jaakko.tyrmi@oulu.fi	Clinical Groups	Women's Health and Reproduction Group
Heidi Silven	University of Oulu, Oulu, Finland	heidi.silven@student.oulu.fi	Clinical Groups	Women's Health and Reproduction Group
Eeva Sliz	University of Oulu, Oulu, Finland	eeva.sliz@oulu.fi	Clinical Groups	Women's Health and Reproduction Group
Riikka Arffman	University of Oulu, Oulu, Finland	riikka.arffman@oulu.fi	Clinical Groups	Women's Health and Reproduction Group
Susanna Savukoski	University of Oulu, Oulu, Finland	susanna.savukoski@oulu.fi	Clinical Groups	Women's Health and Reproduction Group
Triin Laisk	Estonian biobank, Tartu, Estonia	triin.laisk@ut.ee	Clinical Groups	Women's Health and Reproduction Group
Natalia Pujol	Estonian biobank, Tartu, Estonia	natalia.pujolgualdo@oulu.fi	Clinical Groups	Women's Health and Reproduction Group
Mengzhen Liu	Abbvie, Chicago, IL, United States	mengzhen.liu@abbvie.com	Clinical Groups	Women's Health and Reproduction Group
Bridget Riley-Gillis	Abbvie, Chicago, IL, United States	bridget.rileygillis@abbvie.com	Clinical Groups	Women's Health and Reproduction Group
Rion Pendergrass	Genentech, San Francisco, CA, United States	penders2@gene.com	Clinical Groups	Women's Health and Reproduction Group
Janet Kumar	GlaxoSmithKline, Collegeville, PA, United States	janet.x.kumar@gsk.com	Clinical Groups	Women's Health and Reproduction Group
Kirsi Auro	GlaxoSmithKline, Espoo, Finland	kirsi.m.auro@gsk.com	Clinical Groups	Women's Health and Reproduction Group
Iiris Hovatta	University of Helsinki, Finland	iiris.hovatta@helsinki.fi	Clinical Groups	Depression group
Chia-Yen Chen	Biogen, Cambridge, MA, United States	chiayen.chen@biogen.com	Clinical Groups	Depression group
Erkki Isometsä	Hospital District of Helsinki and Uusimaa, Helsinki, Finland	erkki.isometsa@hus.fi	Clinical Groups	Depression group
Hanna Ollila	Institute for Molecular Medicine Finland (FIMM), HiLIFE, University of Helsinki, Helsink	hanna.m.ollila@helsinki.fi	Clinical Groups	Depression group
Jaana Suvisaari	Finnish Institute for Health and Welfare (THL), Helsinki, Finland	jaana.suvisaari@thl.fi	Clinical Groups	Depression group
Thomas Damm Als	Aarhus University, Denmark	tda@biomed.au.dk	Clinical Groups	Depression group
Antti Mäkitie	Department of Otorhinolaryngology - Head and Neck Surgery, University of Helsinki and Helsinki University Hospital, Helsinki, Finland	antti.makitie@helsinki.fi	Clinical Groups	ENT (ear, nose and throath) Group
Argyro Bizaki-Vallaskang	Pirkanmaa Hospital District, Tampere, Finland	argyro.bizaki-vallaskangas@tuni.fi	Clinical Groups	ENT (ear, nose and throath) Group
Sanna Toppila-Salmi	University of Eastern Finland and Kuopio University Hospital, Department of Otorhinolaryngology, Kuopio, Finland and Department of Allergy, Helsinki University Hospital and University of Helsinki, Finland	sanna.salmi@helsinki.fi	Clinical Groups	ENT (ear, nose and throath) Group
Tytti Willberg	Hospital District of Southwest Finland, Turku, Finland	tytti.willberg@tyks.fi	Clinical Groups	ENT (ear, nose and throath) Group
Elmo Saarentaus	Institute for Molecular Medicine Finland (FIMM), HiLIFE, University of Helsinki, Helsink	elmo.saarentaus@helsinki.fi	Clinical Groups	ENT (ear, nose and throath) Group
Antti Aarnisalo	Hospital District of Helsinki and Uusimaa, Helsinki, Finland	antti.aarnisalo@hus.fi	Clinical Groups	ENT (ear, nose and throath) Group
Eveliina Salminen	Hospital District of Helsinki and Uusimaa, Helsinki, Finland	eveliina.e.salminen@hus.fi	Clinical Groups	ENT (ear, nose and throath) Group
Elisa Rahikkala	Northern Ostrobothnia Hospital District, Oulu, Finland	elisa.rahikkala@ppshp.fi	Clinical Groups	ENT (ear, nose and throath) Group
Johannes Kettunen	Northern Ostrobothnia Hospital District, Oulu, Finland	johannes.kettunen@oulu.fi	Clinical Groups	ENT (ear, nose and throath) Group
Kristiina Aittomäki	Department of Medical Genetics, Helsinki University Central Hospital, Helsinki, Finland	kristiina.aittomaki@helsinki.fi	Clinical Groups	POI (premature ovarian failure) Group
Fredrik Åberg	Transplantation and Liver Surgery Clinic, Helsinki University Hospital, Helsinki University, Helsinki, Finland	fredrik.aberg@helsinki.fi	Clinical Groups	LiverScore Group
Mitja Kurki	Institute for Molecular Medicine Finland (FIMM), HiLIFE, University of Helsinki, Helsinki, Finland; Broad Institute, Cambridge, MA, United States	mkurki@broadinstitute.org	FinnGen Analysis working g	FinnGen Analysis working group

Samuli Ripatti	Institute for Molecular Medicine Finland (FIMM), HiLIFE, University of Helsinki, Helsink	samuli.ripatti@helsinki.fi	FinnGen Analysis working gr	FinnGen Analysis working group
Mark Daly	Institute for Molecular Medicine, Finland (FIMM), HiLIFE, University of Helsinki, Helsinki, Finland; Broad Institute of MIT and Harvard; Massachusetts General Hospital	mark.daly@helsinki.fi	FinnGen Analysis working gr	FinnGen Analysis working group
Juha Karjalainen	Institute for Molecular Medicine Finland (FIMM), HiLIFE, University of Helsinki, Helsink	juha.karjalainen@helsinki.fi	FinnGen Analysis working gr	FinnGen Analysis working group
Aki Havulinna	Institute for Molecular Medicine Finland (FIMM), HiLIFE, University of Helsinki, Helsinki,	aki.havulinna@helsinki.fi	FinnGen Analysis working gr	FinnGen Analysis working group
Juha Mehtonen	Institute for Molecular Medicine Finland (FIMM), HiLIFE, University of Helsinki, Helsink	juha.mehtonen@helsinki.fi	FinnGen Analysis working gr	FinnGen Analysis working group
Priit Palta	Institute for Molecular Medicine Finland (FIMM), HiLIFE, University of Helsinki, Helsink	priit.palta@helsinki.fi	FinnGen Analysis working gr	FinnGen Analysis working group
Shabbeer Hassan	Institute for Molecular Medicine Finland (FIMM), HiLIFE, University of Helsinki, Helsink	shabbeer.hassan@helsinki.fi	FinnGen Analysis working gr	FinnGen Analysis working group
Pietro Della Briotta Parol	Institute for Molecular Medicine Finland (FIMM), HiLIFE, University of Helsinki, Helsink	pietro.dellabriottaparolo@helsinki.fi	FinnGen Analysis working gr	FinnGen Analysis working group
Wei Zhou	Broad Institute, Cambridge, MA, United States	wzhou@broadinstitute.org	FinnGen Analysis working gr	FinnGen Analysis working group
Mutaamba Maasha	Broad Institute, Cambridge, MA, United States	mmaasha@broadinstitute.org	FinnGen Analysis working gr	FinnGen Analysis working group
Shabbeer Hassan	Institute for Molecular Medicine Finland (FIMM), HiLIFE, University of Helsinki, Helsink	shabbeer.hassan@helsinki.fi	FinnGen Analysis working gr	FinnGen Analysis working group
Susanna Lemmelä	Institute for Molecular Medicine Finland (FIMM), HiLIFE, University of Helsinki, Helsink	susanna.lemmela@helsinki.fi	FinnGen Analysis working gr	FinnGen Analysis working group
Manuel Rivas	University of Stanford, Stanford, CA, United States	mrivas@stanford.edu	FinnGen Analysis working gr	FinnGen Analysis working group
Aarno Palotie	Institute for Molecular Medicine Finland (FIMM), HiLIFE, University of Helsinki, Helsink	aarno.palotie@helsinki.fi	FinnGen Analysis working gr	FinnGen Analysis working group
Aoxing Liu	Institute for Molecular Medicine Finland (FIMM), HiLIFE, University of Helsinki, Helsink	aoxing.liu@helsinki.fi	FinnGen Analysis working gr	FinnGen Analysis working group
Arto Lehisto	Institute for Molecular Medicine Finland (FIMM), HiLIFE, University of Helsinki, Helsink	arto.lehisto@helsinki.fi	FinnGen Analysis working gr	FinnGen Analysis working group
Andrea Ganna	Institute for Molecular Medicine Finland (FIMM), HiLIFE, University of Helsinki, Helsink	aganna@broadinstitute.org	FinnGen Analysis working gr	FinnGen Analysis working group
Vincent Llorens	Institute for Molecular Medicine Finland (FIMM), HiLIFE, University of Helsinki, Helsink	vincent.llorens@helsinki.fi	FinnGen Analysis working gr	FinnGen Analysis working group
Hannele Laivuori	Institute for Molecular Medicine Finland (FIMM), HiLIFE, University of Helsinki, Helsink	hannele.laivuori@helsinki.fi	FinnGen Analysis working gr	FinnGen Analysis working group
Taru Tukiainen	Institute for Molecular Medicine Finland (FIMM), HiLIFE, University of Helsinki, Helsink	taru.tukiainen@helsinki.fi	FinnGen Analysis working gr	FinnGen Analysis working group
Mary Pat Reeve	Institute for Molecular Medicine Finland (FIMM), HiLIFE, University of Helsinki, Helsink	mary.reeve@helsinki.fi	FinnGen Analysis working gr	FinnGen Analysis working group
Henrike Heyne	Institute for Molecular Medicine Finland (FIMM), HiLIFE, University of Helsinki, Helsink	hheyne@broadinstitute.org	FinnGen Analysis working gr	FinnGen Analysis working group
Nina Mars	Institute for Molecular Medicine Finland (FIMM), HiLIFE, University of Helsinki, Helsink	nina.mars@helsinki.fi	FinnGen Analysis working gr	FinnGen Analysis working group
Joel Rämö	Institute for Molecular Medicine Finland (FIMM), HiLIFE, University of Helsinki, Helsink	joel.ramo@helsinki.fi	FinnGen Analysis working gr	FinnGen Analysis working group
Elmo Saarentaus	Institute for Molecular Medicine Finland (FIMM), HiLIFE, University of Helsinki, Helsink	elmo.saarentaus@helsinki.fi	FinnGen Analysis working gr	FinnGen Analysis working group
Hanna Ollila	Institute for Molecular Medicine Finland (FIMM), HiLIFE, University of Helsinki, Helsink	hanna.m.ollila@helsinki.fi	FinnGen Analysis working gr	FinnGen Analysis working group
Rodos Rodosthenous	Institute for Molecular Medicine Finland (FIMM), HiLIFE, University of Helsinki, Helsink	rodos.rodosthenous@helsinki.fi	FinnGen Analysis working gr	FinnGen Analysis working group
Satu Strausz	Institute for Molecular Medicine Finland (FIMM), HiLIFE, University of Helsinki, Helsink	satu.strausz@helsinki.fi	FinnGen Analysis working gr	FinnGen Analysis working group
Tuula Palotie	University of Helsinki and Hospital District of Helsinki and Uusimaa, Helsinki, Finland	tuula.palotie@helsinki.fi	FinnGen Analysis working gr	FinnGen Analysis working group
Kimmo Palin	University of Helsinki, Helsinki, Finland	kimmo.palin@helsinki.fi	FinnGen Analysis working gr	FinnGen Analysis working group
Javier Garcia-Tabuenca	University of Tampere, Tampere, Finland	javier.graciatabuenca@tuni.fi	FinnGen Analysis working gr	FinnGen Analysis working group
Harri Siirtola	University of Tampere, Tampere, Finland	harri.siirtola@tuni.fi	FinnGen Analysis working gr	FinnGen Analysis working group
Tuomo Kiiskinen	Institute for Molecular Medicine Finland (FIMM), HiLIFE, University of Helsinki, Helsink	tuomo.kiiskinen@helsinki.fi	FinnGen Analysis working gr	FinnGen Analysis working group
Jiwoo Lee	Institute for Molecular Medicine Finland (FIMM), HiLIFE, University of Helsinki, Helsinki, Finland; Broad Institute, Cambridge, MA, United States	jiwoo.lee@helsinki.fi	FinnGen Analysis working gr	FinnGen Analysis working group
Kristin Tsuo	Institute for Molecular Medicine Finland (FIMM), HiLIFE, University of Helsinki, Helsinki, Finland; Broad Institute, Cambridge, MA, United States	kristintsuo@fas.harvard.edu	FinnGen Analysis working gr	FinnGen Analysis working group
Amanda Elliott	Institute for Molecular Medicine Finland (FIMM), HiLIFE, University of Helsinki, Helsinki, Finland; Broad Institute, Cambridge, MA, USA and Massachusetts General Hospital, Boston, MA, USA	aelliott@broadinstitute.org	FinnGen Analysis working gr	FinnGen Analysis working group
Kati Kristiansson	THL Biobank / Finnish Institute for Health and Welfare (THL), Helsinki, Finland	kati.kristiansson@thl.fi	FinnGen Analysis working gr	FinnGen Analysis working group
Mikko Arvas	Finnish Red Cross Blood Service / Finnish Hematology Registry and Clinical Biobank, Helsinki, Finland	mikko.arvas@veripalvelu.fi	FinnGen Analysis working gr	FinnGen Analysis working group
Kati Hyvärinen	Finnish Red Cross Blood Service, Helsinki, Finland	kati.hyvarinen@veripalvelu.fi	FinnGen Analysis working gr	FinnGen Analysis working group
Jarmo Ritari	Finnish Red Cross Blood Service, Helsinki, Finland	jarmo.ritari@veripalvelu.fi	FinnGen Analysis working gr	FinnGen Analysis working group
Olli Carpén	Helsinki Biobank / Helsinki University and Hospital District of Helsinki and Uusimaa, Helsinki	olli.carpen@helsinki.fi	FinnGen Analysis working gr	FinnGen Analysis working group
Johannes Kettunen	Northern Finland Biobank Borealis / University of Oulu / Northern Ostrobothnia Hospital District, Oulu, Finland	johannes.kettunen@oulu.fi	FinnGen Analysis working gr	FinnGen Analysis working group
Katri Pylkäs	University of Oulu, Oulu, Finland	katri.pylkas@oulu.fi	FinnGen Analysis working gr	FinnGen Analysis working group
Eeva Sliz	University of Oulu, Oulu, Finland	eeva.sliz@oulu.fi	FinnGen Analysis working gr	FinnGen Analysis working group
Minna Karjalainen	University of Oulu, Oulu, Finland	minna.k.karjalainen@oulu.fi	FinnGen Analysis working gr	FinnGen Analysis working group
Tuomo Mantere	Northern Finland Biobank Borealis / University of Oulu / Northern Ostrobothnia Hospital District, Oulu, Finland	tuomo.mantere@oulu.fi	FinnGen Analysis working gr	FinnGen Analysis working group
Eeva Kangasniemi	Finnish Clinical Biobank Tampere / University of Tampere / Pirkanmaa Hospital District, Tampere, Finland	eeva.kangasniemi@pshp.fi	FinnGen Analysis working gr	FinnGen Analysis working group
Sami Heikkinen	University of Eastern Finland, Kuopio, Finland	sami.heikkinen@uef.fi	FinnGen Analysis working gr	FinnGen Analysis working group
Arto Mannermaa	Biobank of Eastern Finland / University of Eastern Finland / Northern Savo Hospital District, Kuopio, Finland	arto.mannermaa@uef.fi	FinnGen Analysis working gr	FinnGen Analysis working group
Eija Laakkonen	University of Jyväskylä, Jyväskylä, Finland	eija.k.laakkonen@jyu.fi	FinnGen Analysis working gr	FinnGen Analysis working group
Nina Pitkänen	Auria Biobank / University of Turku / Hospital District of Southwest Finland, Turku, Finland	Niina.Pitkanen@tyks.fi	FinnGen Analysis working gr	FinnGen Analysis working group
Samuel Lessard	Translational Sciences, Sanofi R&D, Framingham, MA, USA	samuel.lessard@sanofi.com	FinnGen Analysis working gr	FinnGen Analysis working group
Clément Chatelain	Translational Sciences, Sanofi R&D, Framingham, MA, USA	clement.chatelain@sanofi.com	FinnGen Analysis working gr	FinnGen Analysis working group
Lila Kallio	Auria Biobank / University of Turku / Hospital District of Southwest Finland, Turku, Finland	Lila.Kallio@tyks.fi	Biobank directors	Biobank directors
Tiina Wahlfors	THL Biobank / Finnish Institute for Health and Welfare (THL), Helsinki, Finland	tiina.wahlfors@thl.fi	Biobank directors	Biobank directors
Jukka Partanen	Finnish Red Cross Blood Service / Finnish Hematology Registry and Clinical Biobank, Helsinki, Finland	jukka.partanen@veripalvelu.fi	Biobank directors	Biobank directors
Eero Punkka	Helsinki Biobank / Helsinki University and Hospital District of Helsinki and Uusimaa, Helsinki	eero.punkka@hus.fi	Biobank directors	Biobank directors
Raisa Serpi	Northern Finland Biobank Borealis / University of Oulu / Northern Ostrobothnia Hospital District, Oulu, Finland	raisa.serpi@ppshp.fi	Biobank directors	Biobank directors
Sanna Siltanen	Finnish Clinical Biobank Tampere / University of Tampere / Pirkanmaa Hospital District, Tampere, Finland	sanna.siltanen@pshp.fi	Biobank directors	Biobank directors
Veli-Matti Kosma	Biobank of Eastern Finland / University of Eastern Finland / Northern Savo Hospital District, Kuopio, Finland	veli-matti.kosma@uef.fi	Biobank directors	Biobank directors
Teijo Kuopio	Central Finland Biobank / University of Jyväskylä / Central Finland Health Care District, Jyväskylä, Finland	teijo.kuopio@ksshp.fi	Biobank directors	Biobank directors
Anu Jalanko	Institute for Molecular Medicine Finland (FIMM), HiLIFE, University of Helsinki, Helsink	anu.jalanko@helsinki.fi	FinnGen Teams	Administration
Huei-Yi Shen	Institute for Molecular Medicine Finland (FIMM), HiLIFE, University of Helsinki, Helsink	huei-yi.shen@helsinki.fi	FinnGen Teams	Administration
Risto Kajanne	Institute for Molecular Medicine Finland (FIMM), HiLIFE, University of Helsinki, Helsink	risto.kajanne@helsinki.fi	FinnGen Teams	Administration
Mervi Aavikko	Institute for Molecular Medicine Finland (FIMM), HiLIFE, University of Helsinki, Helsink	mervi.aavikko@helsinki.fi	FinnGen Teams	Administration
Helen Cooper	Institute for Molecular Medicine Finland (FIMM), HiLIFE, University of Helsinki, Helsink	helen.cooper@helsinki.fi	FinnGen Teams	Administration
Denise Öller	Institute for Molecular Medicine Finland (FIMM), HiLIFE, University of Helsinki, Helsink	denise.oller@helsinki.fi	FinnGen Teams	Administration
Rasko Leinonen	Institute for Molecular Medicine Finland (FIMM), HiLIFE, University of Helsinki, Helsink	rasko@ebi.ac.uk	FinnGen Teams	Administration
Henna Palin	Finnish Clinical Biobank Tampere / University of Tampere / Pirkanmaa Hospital District, Tampere, Finland	henna.palin@pshp.fi	FinnGen Teams	Administration
Malla-Maria Linna	Helsinki Biobank / Helsinki University and Hospital District of Helsinki and Uusimaa, Helsinki	malla-maria.linna@hus.fi	FinnGen Teams	Administration
Mitja Kurki	Institute for Molecular Medicine Finland (FIMM), HiLIFE, University of Helsinki, Helsinki, Finland; Broad Institute, Cambridge, MA, United States	mkurki@broadinstitute.org	FinnGen Teams	Analysis
Juha Karjalainen	Institute for Molecular Medicine Finland (FIMM), HiLIFE, University of Helsinki, Helsink	juha.karjalainen@helsinki.fi	FinnGen Teams	Analysis
Pietro Della Briotta Parol	Institute for Molecular Medicine Finland (FIMM), HiLIFE, University of Helsinki, Helsink	pietro.dellabriottaparolo@helsinki.fi	FinnGen Teams	Analysis
Arto Lehisto	Institute for Molecular Medicine Finland (FIMM), HiLIFE, University of Helsinki, Helsink	arto.lehisto@helsinki.fi	FinnGen Teams	Analysis
Juha Mehtonen	Institute for Molecular Medicine Finland (FIMM), HiLIFE, University of Helsinki, Helsink	juha.mehtonen@helsinki.fi	FinnGen Teams	Analysis
Wei Zhou	Broad Institute, Cambridge, MA, United States	wzhou@broadinstitute.org	FinnGen Teams	Analysis
Masahiro Kanai	Broad Institute, Cambridge, MA, United States	mkanai@broadinstitute.org	FinnGen Teams	Analysis
Mutaamba Maasha	Broad Institute, Cambridge, MA, United States	mmaasha@broadinstitute.org	FinnGen Teams	Analysis
Zhili Zheng	Broad Institute, Cambridge, MA, United States	zhengzhi@broadinstitute.org	FinnGen Teams	Analysis
Hannele Laivuori	Institute for Molecular Medicine Finland (FIMM), HiLIFE, University of Helsinki, Helsink	hannele.laivuori@helsinki.fi	FinnGen Teams	Clinical Endpoint Development
Aki Havulinna	Institute for Molecular Medicine Finland (FIMM), HiLIFE, University of Helsinki, Helsinki,	aki.havulinna@helsinki.fi	FinnGen Teams	Clinical Endpoint Development
Susanna Lemmelä	Institute for Molecular Medicine Finland (FIMM), HiLIFE, University of Helsinki, Helsink	susanna.lemmela@helsinki.fi	FinnGen Teams	Clinical Endpoint Development
Tuomo Kiiskinen	Institute for Molecular Medicine Finland (FIMM), HiLIFE, University of Helsinki, Helsink	tuomo.kiiskinen@helsinki.fi	FinnGen Teams	Clinical Endpoint Development
L. Elisa Lahtela	Institute for Molecular Medicine Finland (FIMM), HiLIFE, University of Helsinki, Helsink	laura.lahtela@helsinki.fi	FinnGen Teams	Clinical Endpoint Development
Mari Kaunisto	Institute for Molecular Medicine Finland (FIMM), HiLIFE, University of Helsinki, Helsink	mari.kaunisto@helsinki.fi	FinnGen Teams	Communication
Elina Kilpeläinen	Institute for Molecular Medicine Finland (FIMM), HiLIFE, University of Helsinki, Helsink	elina.kilpelainen@helsinki.fi	FinnGen Teams	E-Science
Timo P. Sipilä	Institute for Molecular Medicine Finland (FIMM), HiLIFE, University of Helsinki, Helsink	timo.p.sipila@helsinki.fi	FinnGen Teams	E-Science
Oluwaseun Alexander Da	Institute for Molecular Medicine Finland (FIMM), HiLIFE, University of Helsinki, Helsink	alexander.dada@helsinki.fi	FinnGen Teams	E-Science
Awaisa Ghazal	Institute for Molecular Medicine Finland (FIMM), HiLIFE, University of Helsinki, Helsink	awaisa.ghazal@helsinki.fi	FinnGen Teams	E-Science
Anastasia Kytölä	Institute for Molecular Medicine Finland (FIMM), HiLIFE, University of Helsinki, Helsink	anastasia.shcherban@helsinki.fi	FinnGen Teams	E-Science
Rigbe Weldatsadik	Institute for Molecular Medicine Finland (FIMM), HiLIFE, University of Helsinki, Helsink	rigbe.weldatsadik@helsinki.fi	FinnGen Teams	E-Science
Sanni Ruotsalainen	Institute for Molecular Medicine Finland (FIMM), HiLIFE, University of Helsinki, Helsink	sanni.ruotsalainen@helsinki.fi	FinnGen Teams	E-Science
Kati Donner	Institute for Molecular Medicine Finland (FIMM), HiLIFE, University of Helsinki, Helsink	kati.donner@helsinki.fi	FinnGen Teams	Genotyping
Timo P. Sipilä	Institute for Molecular Medicine Finland (FIMM), HiLIFE, University of Helsinki, Helsink	timo.p.sipila@helsinki.fi	FinnGen Teams	Genotyping
Anu Loukola	Helsinki Biobank / Helsinki University and Hospital District of Helsinki and Uusimaa, Helsinki	anu.loukola@hus.fi	FinnGen Teams	Sample Collection Coordination
Päivi Laiho	THL Biobank / Finnish Institute for Health and Welfare (THL), Helsinki, Finland	paivi.laiho@thl.fi	FinnGen Teams	Sample Logistics
Tuuli Sistonen	THL Biobank / Finnish Institute for Health and Welfare (THL), Helsinki, Finland	tuuli.sistonen@thl.fi	FinnGen Teams	Sample Logistics

Essi Kaiharju	THL Biobank / Finnish Institute for Health and Welfare (THL), Helsinki, Finland	essi.kaiharju@thl.fi	**FinnGen Teams**	**Sample Logistics**
Markku Laukkanen	THL Biobank / Finnish Institute for Health and Welfare (THL), Helsinki, Finland	markku.laukkanen@thl.fi	**FinnGen Teams**	**Sample Logistics**
Elina Järvensivu	THL Biobank / Finnish Institute for Health and Welfare (THL), Helsinki, Finland	elina.jarvensivu@thl.fi	**FinnGen Teams**	**Sample Logistics**
Sini Lähteenmäki	THL Biobank / Finnish Institute for Health and Welfare (THL), Helsinki, Finland	sini.lahteenmaki@thl.fi	**FinnGen Teams**	**Sample Logistics**
Lotta Männikkö	THL Biobank / Finnish Institute for Health and Welfare (THL), Helsinki, Finland	lotta.mannikko@thl.fi	**FinnGen Teams**	**Sample Logistics**
Regis Wong	THL Biobank / Finnish Institute for Health and Welfare (THL), Helsinki, Finland	regis.wong@thl.fi	**FinnGen Teams**	**Sample Logistics**
Auli Toivola	THL Biobank / Finnish Institute for Health and Welfare (THL), Helsinki, Finland	auli.toivola@thl.fi	**FinnGen Teams**	**Sample Logistics**
Minna Brunfeldt	THL Biobank / Finnish Institute for Health and Welfare (THL), Helsinki, Finland	minna.brunfeldt@thl.fi	**FinnGen Teams**	**Registry Data Operations**
Hannele Mattsson	THL Biobank / Finnish Institute for Health and Welfare (THL), Helsinki, Finland	hannele.mattsson@thl.fi	**FinnGen Teams**	**Registry Data Operations**
Kati Kristiansson	THL Biobank / Finnish Institute for Health and Welfare (THL), Helsinki, Finland	kati.kristiansson@thl.fi	**FinnGen Teams**	**Registry Data Operations**
Susanna Lemmelä	Institute for Molecular Medicine Finland (FIMM), HiLIFE, University of Helsinki, Helsink	susanna.lemmela@helsinki.fi	**FinnGen Teams**	**Registry Data Operations**
Sami Koskelainen	THL Biobank / Finnish Institute for Health and Welfare (THL), Helsinki, Finland	sami.koskelainen@thl.fi	**FinnGen Teams**	**Registry Data Operations**
Tero Hiekkalinna	THL Biobank / Finnish Institute for Health and Welfare (THL), Helsinki, Finland	tero.hiekkalinna@helsinki.fi	**FinnGen Teams**	**Registry Data Operations**
Teemu Paajanen	THL Biobank / Finnish Institute for Health and Welfare (THL), Helsinki, Finland	teemu.paajanen@thl.fi	**FinnGen Teams**	**Registry Data Operations**
Priit Palta	Institute for Molecular Medicine Finland (FIMM), HiLIFE, University of Helsinki, Helsink	priit.palta@helsinki.fi	**FinnGen Teams**	**Sequencing Informatics**
Kalle Pärn	Institute for Molecular Medicine Finland (FIMM), HiLIFE, University of Helsinki, Helsink	kalle.parn@helsinki.fi	**FinnGen Teams**	**Sequencing Informatics**
Mart Kals	Institute for Molecular Medicine Finland (FIMM), HiLIFE, University of Helsinki, Helsink	mart.kals@helsinki.fi	**FinnGen Teams**	**Sequencing Informatics**
Shuang Luo	Institute for Molecular Medicine Finland (FIMM), HiLIFE, University of Helsinki, Helsink	shuang.luo@helsinki.fi	**FinnGen Teams**	**Sequencing Informatics**
Tarja Laitinen	Pirkanmaa Hospital District, Tampere, Finland	tarja.laitinen@pshp.fi	**FinnGen Teams**	**Trajectory**
Mary Pat Reeve	Institute for Molecular Medicine Finland (FIMM), HiLIFE, University of Helsinki, Helsink	mary.reeve@helsinki.fi	**FinnGen Teams**	**Trajectory**
Shanmukha Sampath Pa	Institute for Molecular Medicine Finland (FIMM), HiLIFE, University of Helsinki, Helsink	sam.padmanabhuni@helsinki.fi	**FinnGen Teams**	**Trajectory**
Marianna Niemi	University of Tampere, Tampere, Finland	marianna.niemi@tuni.fi	**FinnGen Teams**	**Trajectory**
Harri Siirtola	University of Tampere, Tampere, Finland	harri.siirtola@tuni.fi	**FinnGen Teams**	**Trajectory**
Javier Gracia-Tabuenca	University of Tampere, Tampere, Finland	javier.graciatabuenca@tuni.fi	**FinnGen Teams**	**Trajectory**
Mika Helminen	University of Tampere, Tampere, Finland	mika.helminen@tuni.fi	**FinnGen Teams**	**Trajectory**
Tiina Luukkaala	University of Tampere, Tampere, Finland	tiina.luukkaala@tuni.fi	**FinnGen Teams**	**Trajectory**
Iida Vähätalo	University of Tampere, Tampere, Finland	iida.vahatalo@epshp.fi	**FinnGen Teams**	**Trajectory**
Jyrki Tammerluoto	Institute for Molecular Medicine Finland (FIMM), HiLIFE, University of Helsinki, Helsink	jyrki.tammerluoto@helsinki.fi	**FinnGen Teams**	**Data protection officer**
Marco Hautalahti	Finnish Biobank Cooperative - FINBB	marco.hautalahti@finbb.fi	**FinnGen Teams**	**FINBB - Finnish biobank cooperative**
Johanna Mäkelä	Finnish Biobank Cooperative - FINBB	johanna.makela@finbb.fi	**FinnGen Teams**	**FINBB - Finnish biobank cooperative**
Sarah Smith	Finnish Biobank Cooperative - FINBB	sarah.smith@finbb.fi	**FinnGen Teams**	**FINBB - Finnish biobank cooperative**
Tom Southerington	Finnish Biobank Cooperative - FINBB	tom.southerington@finbb.fi	**FinnGen Teams**	**FINBB - Finnish biobank cooperative**
Petri Lehto	Finnish Biobank Cooperative - FINBB	petri.lehto@finbb.fi	**FinnGen Teams**	**FINBB - Finnish biobank cooperative**
